# Donor-Independent Metabolomics Enables Bloodstain Age Determination at Crime Scenes

**DOI:** 10.1021/acs.jproteome.6c00199

**Published:** 2026-06-19

**Authors:** Kirstine L. Nielsen, Johan K. Lassen, Ida Marie M. Løber, Frederik Skovbo, Kim Frisch, Tomasz P. Czaja, Bekzod Khakimov, Søren B. Engelsen, Mogens Johannsen, Palle Villesen

**Affiliations:** † Department of Forensic Medicine, 1006 Aarhus University, Aarhus 8200, Denmark; ‡ Bioinformatics Research Center, Aarhus University, Aarhus 8000, Denmark; § Department of Food Science, 87017 University of Copenhagen, Copenhagen 1958, Denmark; ∥ Department of Clinical Medicine, Aarhus University, Aarhus 8000, Denmark

**Keywords:** bloodstain, time-since-deposition, metabolomics, biomarker ratios, chemometric modeling, machine learning

## Abstract

Determining when bloodstains were deposited remains an unsolved challenge in forensic science, limiting investigators’ ability to reconstruct events and verify suspect timelines. Here, we develop a metabolomics-based approach combining liquid chromatography–mass spectrometry (LC-MS) with machine learning to estimate bloodstain age independent of donor-specific variation. Through untargeted analysis and degradation studies, we identified 51 time-dependent biomarkers and transformed their intensities into stable ratios that normalize for individual differences and blood volume. Using samples collected under controlled environmental conditions, we achieve high accuracy for forensically relevant timeframes with prediction errors of ∼7 h for fresh bloodstains and near-perfect classification of samples as recent (<60 h) or aged (>60 h). Validation on two independent data sets confirms strong performance under typical indoor conditions, while highlighting sensitivity to extreme environmental fluctuations. By addressing key biological and technical sources of variability that have hindered translation to practice, this study establishes a robust analytical framework for bloodstain age estimation. The approach offers a practical foundation for future operational implementation and has the potential to substantially improve forensic timeline reconstruction.

## Graphical abstract

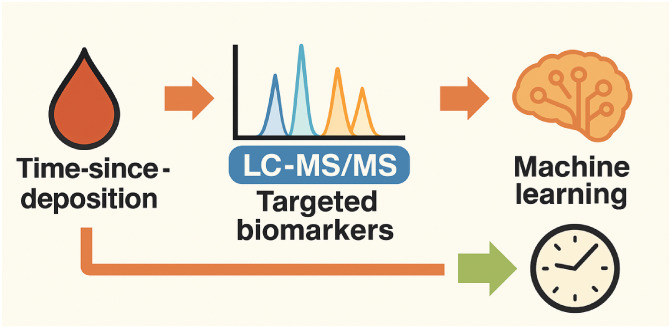

## Introduction

Bloodstains left behind at violent crimes serve as forensic evidence, and ideally, determining whether a bloodstain is recent would greatly aid investigations. Information about the time since deposition (age estimation) could establish the relevance of bloodstains to a specific crime and help set a time frame for the reconstruction of events.^1^

Several age estimation methods, such as optical profiling,^2–4^ hyperspectral imaging,^5,6^ and spectroscopy methods^7–9^ have been tested. These may serve as first and fast onsite screening tools but can be influenced by factors such as surface background and misinformation from biological decay, which may affect certainty and validation in age predictions. As such, metabolites may serve as useful age predictors, being subject to nonenzymatic oxidation and hydrolysis when deposited outside the body.

Seok et al. were the first to use untargeted metabolomics as a proof of concept to successfully identify metabolic markers showing reliable increases or decreases with deposition age for up to 3 weeks in blood.^10,11^ Additional studies show that molecular degradation depends on several covariates, such as light,^12,13^ temperature, and humidity.^14^ The kinetics of these changes may vary by donor, metabolite, and conditions, and thus, one or a few compounds may not suffice for reliable predictions, as biological and environmental variations impact the outcome.

Although bloodstain age estimation has been sought through many different methods over the years, currently, no validated technique has been implemented in forensic practice.^1,15^ In this work, we use untargeted and targeted metabolomics to identify aging biomarkers for the development of time-since-deposition prediction models. We established a three-step protocol to eliminate false positives and unstable biomarkers. The first step involves untargeted analysis of bloodstains to select a panel of potential aging biomarkers, including metabolite-breakdown product pairs, based on unsupervised filtering, random forest modeling, and metabolite breakdown experiments (Figure 1a). The second step uses targeted analysis of bloodstains to train simple linear prediction models based on biomarker ratios that do not require normalization (Figure 1b). Step two also includes removing unstable and false-positive biomarkers from step one using technical replicates, inspired by Kim et al.,^16^ and evaluating temperature and humidity effects. The third step validates the robust biomarker ratios on two external validation data sets (Figure 1c). Our approach addresses environmental influences, interindividual variability, and technical confounders, factors previously investigated but not integrated into the estimation of time-since-deposition of bloodstains. This advances our goal of developing a robust and reliable tool for forensic applications.

**Figure 1:**
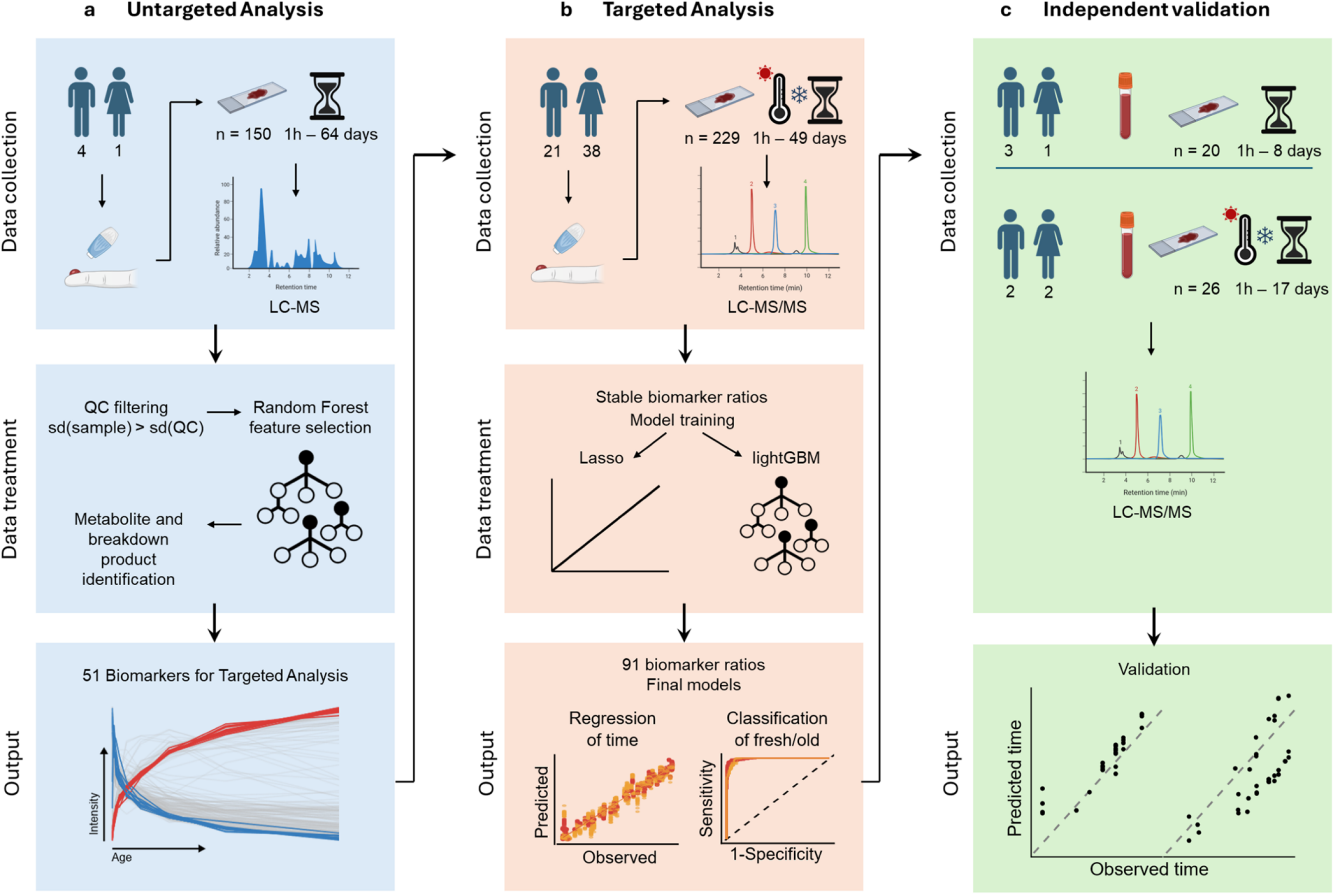
Schematic of the study design. (a) Untargeted analysis of bloodstains (*N* = 5; 10 time points; 3 biological replicates; *n* = 150 samples) to select aging biomarkers. (b) Targeted analysis of bloodstains (*N* = 59; random time points; 5 conditions; *n* = 229 samples) to train simple prediction models to predict time-since-deposition under different conditions. Samples were stored under uncontrolled indoor conditions or at different known temperatures (10, 23, and 30 °C) and humidity levels (20%, 40%, and 70%). (c) Validation using two independent validation sets (*Validation set 1*: *N* = 4; 5 time points; *n* = 20 samples. *Validation set 2*: *N* = 4; random time points; *n* = 26 samples) to test model performance. The extended aging period in (a) and (b) (up to 64 days) was used for exploratory biomarker discovery, whereas later data sets focused on shorter, forensically relevant timeframes following consultation with law enforcement. Data collection information is further specified in Supplementary Table S1.

## Experimental Section

### Chemicals

All chemical standards, including internal standards, were purchased from Sigma-Aldrich (Germany). Methanol (MeOH, LC-MS hypergrade), acetonitrile (MeCN, LC-MS hypergrade), and formic acid were purchased from Merck (Germany). Purified Milli-Q water was prepared using a Milli-Q IQ 7000 system.

### Blood Sample Collection and Aging

Four different data sets were made: an untargeted data set, a targeted data set, and two validation sets. An overview is given in Figure 1 and Supplementary Table S1.

The untargeted data set consisted of blood from five different donors (four males and one female). Blood was donated at 30 different time points of the donor’s own choice by making a fingertip needle prick and placing one drop of whole blood (approximately 50 μL) on a glass slide. The samples were allowed to age for 1 h (the blood was allowed to dry), 6 h, 12 h, 24 h, 2 days, 4 days, 8 days, 16 days, 32 days, and 64 days in an incubator (Termaks KB8400F, Nordic Labtech, Sweden) kept at 23 °C and 40% humidity. The incubator had windows allowing light to come through but was facing north. A total of 15 samples per time point (three per donor) was collected.

The targeted data set consisted of samples from the untargeted data set plus 79 additional samples from 53 different donors (17 males and 36 females), donating between 1 and 7 times independently of each other. These samples were collected using fingertip needle pricks, and the blood volume was not controlled. The samples were additionally divided into several experiments and aged in an incubator (Termaks KB8400F, Nordic Labtech, Sweden). Sixteen samples were kept at 30 °C and 40% humidity, 17 samples were kept at 10 °C and 40% humidity, 10 samples were kept at 23 °C and 70% humidity, and 10 samples were kept at 23 °C and 20% humidity. Finally, 26 samples were kept in different places indoors as mock-up samples (temperature and humidity not measured).

Validation sets 1 and 2 were collected using venipuncture into a 6 mL Vacuette tube with no additive (Greiner Bio-One, Austria). Validation set 1 was collected from 4 donors (3 males and 1 female), each donating once. Validation set 2 was collected from 4 new donors (2 males and 2 females), each donating once. The blood from each donor was pipetted in a volume of 50 μL onto glass plates. Samples from validation set 1 were aged in the incubator at 23 °C and 40% humidity (like the discovery set) and included 20 samples in total (five from each donor). Samples from validation set 2 were aged in different places indoors (temperature and humidity unknown). It included a total of 26 samples (5–8 samples from each donor). The temperature at the time of sampling was measured.

At the end of aging, all the dried blood samples were scraped off the glass with a scalpel and transferred into an Eppendorf tube. The samples were stored at −80 °C for untargeted analysis. For validation sets 1 and 2, we used a storage temperature consistent with a normal freezer (−20 °C) to better reflect practical forensic conditions.

### Extraction and Preparation of Dried Blood Samples for Analysis

An Eppendorf tube with dried blood was added to 600 μL of ice cold 80% MeOH and sonicated for 5 min at room temperature. The mixture was allowed to incubate on ice for 30 min prior to centrifugation at 10,000×g for 5 min. Following centrifugation, 400 μL of the supernatant was transferred to a tube and allowed to evaporate to dryness in a SpeedVac at 35 °C overnight. The dry sample residue was resuspended in 100 μL of Milli-Q water containing 5% MeCN and 0.01% formic acid. For targeted LC-MS/MS analysis, samples were added to an internal standard mix during resuspension, consisting of L-glutamic acid-^13^C_5_, L-tryptophan-D_8_, and dipalmitoyl DL-α-phosphatidylcholine (U–^13^C_40_) to a final concentration of 1, 0.1, and 0.1 μg/mL, respectively.

### Untargeted LC-MS Analysis and Modeling for Selection of Aging Biomarkers

Untargeted analysis was performed using an ACQUITY I-Class UPLC system (Waters Corporation, Milford, MA, USA), coupled to a Bruker maXis Impact QTOF mass spectrometer (Bruker Daltonics, Bremen, Germany).

Chromatographic separation was achieved using an ACQUITY UPLC HSS T3 C18 column (2.1 mm × 100 mm, 1.8 μm, Waters) with gradient elution. Mobile phase A consisted of Milli-Q water with 0.1% formic acid (v/v), and mobile phase B consisted of a 1:1 mixture of MeOH:MeCN with 0.1% formic acid. The gradient started at 0% B from 0 to 2 min, followed by a linear increase of B from 0 to 40% within 2–6 min, 40% to 60% within 6–6.5 min, 60% to 88% within 6.5–11 min, and 88% to 100% within 11–11.5 min. The gradient was then maintained at 100% B for 11.5–17 min. A linear decrease from 100 to 0% B was done from 17 to 18 min, and the column was equilibrated at 0% B from 18 to 21 min before the next injection. The column temperature was set to 50 °C, and the flow rate was 0.4 mL/min. The injection volume was 10 μL, and the sample temperature in the autosampler was maintained at 6 °C.

The mass spectrometer was operated in both positive and negative electrospray ionization (ESI) modes within a mass range of 50–1000 *m*/*z*. The nebulizing gas pressure was 1.2 bar, and the capillary voltage was 4.0 kV in positive ESI and 2.5 kV in negative ESI. The drying gas flow was 8.0 L/min at a temperature of 220 °C. MS scans were acquired in full-scan mode at a sampling rate of 4 Hz. MS/MS scans on selected samples in data-dependent acquisition (DDA) mode (auto-MS/MS) were additionally acquired for compound identification using collision energies of 10, 20, and 30 eV at a sampling rate of 10 Hz. Internal calibration was performed at the end of each chromatographic run using sodium formate.

The untargeted data set was split randomly into two batches and analyzed in both ESI modes. A mix of 37 standard metabolite compounds was analyzed prior to and at the end of each batch to evaluate instrument performance. A quality control (QC) sample was made by pooling equal amounts of each sample within one sample set. The QC sample was injected four times at the beginning of a batch to equilibrate the system. The QC sample was additionally injected between every seven samples for quality assessment of instrument performance during the batch. Satisfactory instrument performance across a batch was achieved based on consistent retention times and base peak intensities across QC injections. The samples were injected at random. A blind sample consisting of water, prepared the same way as the blood samples, was injected at the beginning and the end of each batch.

The MS data were converted to the *mzML* file format using msConvert from ProteoWizard (http://proteowizard.sourceforge.net). The *mzML* files were preprocessed using the XCMS^17^ and CAMERA^18^ packages (R language). The centWave algorithm was applied for peak picking using a resolution of 12 ppm and a signal-to-noise ratio ≥ 6, and peaks were aligned by loess regression and grouped by density-based clustering (bandwidth: 2.5 s). Gap filling was included to recover missing signals in the raw data. Features had to be present in a minimum of 80% of the samples within a sample (age) group.

The preprocessed data resulted in a positive and negative ESI data set containing 13,046 and 3,786 features, respectively. Each ESI data set went through a series of transformations and filters. First, fourth-root transformation normalized the data to an approximate normal distribution. Second, PCA was performed on sample-type observations (centered and scaled, retaining 12 components). A sample was flagged as an outlier on a given PC axis if its score deviated from the axis median by more than 1.5× the 95th-percentile-minus-fifth-percentile range (i.e., |score – median| > 1.5 × (Q95 – Q05)). Samples flagged on one or more axes were excluded. Third, features were removed entirely if any single observation exceeded median +1.5 × Q90 of the given feature’s distribution. This was applied globally across all observations per feature. Fourth, features with either (1) smaller sample or QC intensity than blinds or (2) smaller variance in samples than QCs or blinds were removed. Fifth, data were row-normalized using a reference feature set identified from QC samples by retaining features whose median intensity rank fell within the 20th–80th percentile range, then removing the 20% most variable among these by rank range, yielding a set of stably abundant features. Each sample was normalized by dividing all its feature intensities by the sum of its intensities across this reference feature set, then multiplying by a global scaling constant (the maximum intensity in the prenormalization matrix) to preserve the original intensity scale. After the row-normalization step, step four was repeated, returning the positive and negative data set that contained 2,134 and 519 features, of which the top 250 most variable features were selected for modeling.

Each data set was modeled with random forest using 10 repeated 10-fold cross-validation to evaluate the regression performance (RMSE). To fit the model, the caret^19^ package (R language) was used with a *tune length* of 5 (see Extended Data 2 for grid search results). Feature importance was estimated using the permutation method implemented in the ranger random forest package, where each feature was permuted once per tree, and importance was defined as the mean decrease in out-of-bag (OOB) prediction accuracy across all trees. The top 50 features were selected from each ESI data set. Following this, the features were manually assessed based on intensity, robustness, and quality of annotation to form a final feature list that was used for quantitative metabolomics. Of the 50 selected features in each data set, 10 compounds were selected from positive ESI, and 10 were selected from negative ESI, excluding fragments, isotopes, and multiply charged ions.

### Annotation of Metabolites and Breakdown Products

Metabolite annotation was performed by matching the *m*/*z* value, retention time, and MS/MS fragments with an in-house database (Level 1 identification). Additionally, matching with the publicly available Human Metabolome Database (HMDB) was used for further annotation (Level 2 based on MS/MS fragments or Level 3 based on *m*/*z* value only). Annotation levels can be seen in the Supplementary data S1 Extended Data.

Degradation studies with standard compounds of identified metabolites were performed to identify possible breakdown products. Compounds included in the study were tryptophan, kynurenine, kynurenic acid, ergothioneine, 3-hydroxybutyric acid (BHB), spermidine, palmitoyl carnitine, oxidized glutathione, glutamic acid, cystine, tyrosine, β-nicotinamide adenine dinucleotide (NAD^+^), 1-palmitoyl-2-oleoyl-*sn*-glycero-3-phosphocholine (PC (16:0/18:1)), and 1-stearoyl-2-linoleoyl-*sn*-glycero-3-phosphocholine (PC (18:0/18:2)). The compounds were dissolved at 1 mg/mL in water or MeOH, depending on polarity. A volume of 10 μL of each solution, corresponding to 10 μg, was placed on a glass slide and left in the incubator (Termaks KB8400F, Nordic Labtech, Sweden) at 23 °C and 40% humidity for 1 h, 24 h, 7 days, and 14 days. Following incubation, the compounds were flushed off the glass slide with 80% MeOH into a tube and evaporated to dryness in a SpeedVac at 35 °C. The residue was dissolved in 100 μL of Milli-Q water containing 5% MeCN and 0.01% formic acid. The samples were analyzed using the above untargeted LC-MS analysis, and the chromatograms were examined for possible newly appearing peaks.

### Targeted LC-MS/MS Analysis of Aging Biomarkers

Semiquantitative analysis of 51 targeted compounds was performed on a Sciex ExionLC with a Sciex QTRAP 6500+. The chromatographic conditions were similar to the untargeted LC-MS analysis. Direct infusion of single standards for the known metabolite compounds was utilized to optimize multiple reaction monitoring (MRM) parameters, whereas the conditions for unknown breakdown products were based on MS/MS measurements performed in the untargeted LC-MS analysis. The MRM conditions can be found in Supplementary Table S2. The QTRAP was operated in both positive and negative ESI modes, with a probe temperature of 500 °C and an ion spray voltage of 4.5 kV in ESI+ and −4.5 kV in ESI–. The pressures of the curtain gas (CUR), ion source gas 1 (GS1), ion source gas 2 (GS2), and collision gas (CAD) were set at 20, 60, 50, and 9 psi, respectively. Nitrogen was used as the CAD gas.

We analyzed all data sets independently using the method. A mix of 17 standard metabolite compounds included in the analysis (see Supplementary Table S2) was analyzed at the beginning and end of each batch to evaluate instrument performance. Samples were analyzed in random order. An interbatch quality assurance sample was prepared by pooling extracts from 24-h-old blood samples and analyzed with each batch. Additionally, we made a quality control (QC) sample by pooling equal amounts of each sample within one sample set and analyzed it continuously, as in the untargeted LC-MS setup. We used isotopically labeled internal standards in the samples (except for the untargeted data set) to monitor analytical variability. Finally, we reanalyzed 105 randomly selected samples interbatch to create technical replicates.

### Data Analysis and Machine Learning Modeling of Targeted LC-MS/MS Data

For technical replicates (samples being analyzed in duplicate interbatch), we expect each biomarker to have the same intensity for both runs. We calculated all possible pairwise ratios of the biomarkers and then used all technical replicates (several samples injected twice within or between batches) to select ratios that showed both high biological variation (variation between samples of different ages and conditions) and low technical variation (variation between replicates). We calculated the Pearson correlation between replicate 1 and replicate 2 across all technical replicates for each ratio. We calculated the ratio as log2­[(feature1 + 1)/(feature2 + 1)] and only kept ratios with Pearson *r* > 0.95. (If a specific ratio is truly identical in all samples, measured differences will only be random noise with an expected correlation around zero.).The selected ratios were then calculated for all data sets before machine learning.

We first fitted two regression models: a regularized linear model (least absolute shrinkage and selection operator, Lasso) and a more flexible model (light gradient boosting machine, LightGBM), to the stable ratio data. For all machine learning, we used repeated cross-validation to test model performance. Each test fold (10% of the data) was only evaluated using a model fitted to the training fold (90% of the data). We repeated this procedure 20 times to estimate how much the performance changed for different train/test splits. We tuned the Lasso models (the regularization parameter) using internal cross-validation on the training fold (10-fold CV). LightGBM was used with default parameters, except that we used 300 iterations and a learning rate of 0.01.

We estimated the effect of temperature by comparing models trained on samples stored at 23 °C and then predicting on samples stored at 10, 23, and 30 °C. We converted time and temperature to “degree-hours” (temperature*time) and used that for modeling to minimize temperature effects.

We estimated the effect of humidity by comparing the performance of models trained on samples stored at 40% humidity and then predicting their performance on samples stored at both 40% and 70% RH.

For the regression models, we used log1p (degree-hours) as a response, both to account for heteroscedasticity and the fact that time is non-negative. We calculated R^2^ values using Pearson correlation between predicted and observed values and tested model differences using the Mann–Whitney U test on the R^2^ values.

Second, we applied Lasso and LightGBM classification models, using time since deposition as the response variable. The models were trained to predict the probability that a given sample exceeded 60 h, thereby enabling binary classification of bloodstains as either recent (<60 h) or aged (>60 h). We converted the probability to symmetric log-odds ratios (log10­(*p*/1 – *p*)) for easier interpretation and visualization of individual probabilities. AUCs for different machine learning models were compared using the Mann–Whitney U test.

## Results and Discussion

### Selection of Biomarkers in Bloodstains Reflecting Time-Since-Deposition

The untargeted LC-MS metabolomics analysis yielded 3786 (negative ESI) and 13046 (positive ESI) features across 150 samples in negative and positive electrospray ionization (ESI) modes, respectively, after preprocessing. To increase the prediction power, we performed data cleaning based on outlier detection and unsupervised feature variance metrics, comparing pooled QCs, samples, and blanks, resulting in 239 (neg ESI) and 250 (pos ESI) features. Principal component analysis (PCA) revealed that age was the biggest source of variance both before (Figure 2a) and after data cleaning (Figure 2b). However, the cleaning resulted in an increase from 31% to 71% in explained variance in PC1; thus, the variance related to age was conserved in the reduced data. Also, the clusters appearing before data cleaning (batch effect, Supplementary Figure S1) were removed in this process. The heatmap of intensities from the top 250 features (pos ESI, Figure 2c) shows two dominant feature clusters, where the intensity for the features on the left side seems to increase from fresh to old, whereas a decrease is observed in the middle/right side (Figure 2c). These clusters indicate clear formation and degradation signals over time, highlighting the efficiency of the unsupervised filtering process. The remaining blocks of clusters are small and show more complex or random patterns. The negative ESI data have similar quality to the positive data, and PCA and heatmaps before cleaning are presented in Supplementary Figures S2–S4.

**Figure 2:**
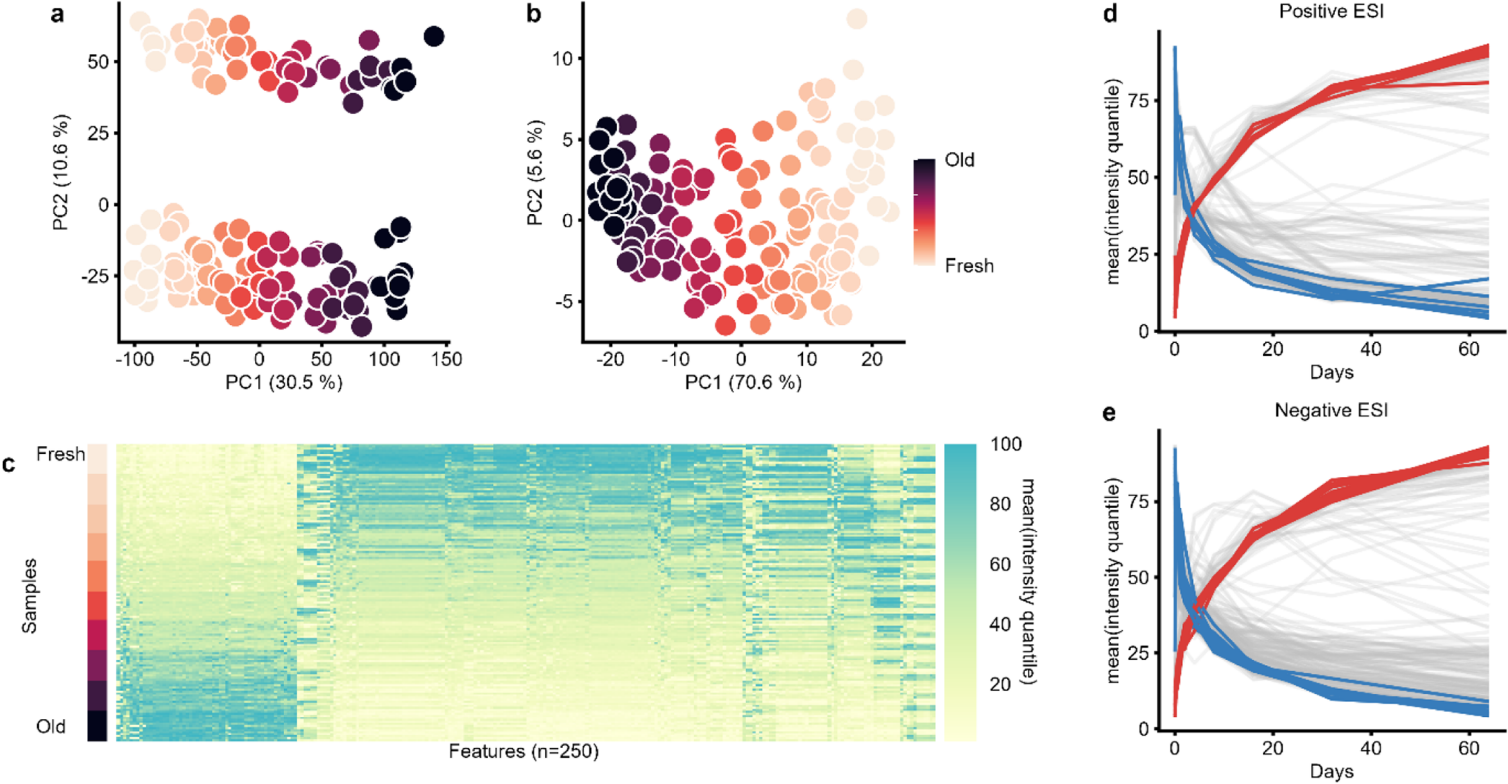
Selection of aging biomarkers in untargeted metabolomics data. (a) PCA-scores plot of 150 bloodstains in positive ESI before normalization and unsupervised data filtering, with color indicating sample age. PC1 describes 31% of the total variance connected to time-since-deposition. (b) PCA-scores plot after normalization and filtering in positive ESI. PC1 describes 71% of the variance, and confounding effects are removed on PC2. (c) Heat map of features from (b) in positive ESI (*y*-axis) clustered by complete hierarchical clustering (columns) and arranged by sample age (rows, fresh to old). (d, e) Random Forest-selected features (red: increasing; blue: decreasing) in positive and negative ESI, nonselected features shown in gray.

We then fitted a random forest regression model to predict time since deposition and chose the top 50 predictive features in both positive and negative ESI for identification. Several of the features increased in concentration with time, as also observed in the heatmap, indicating that they are degradation products of metabolites or proteins formed during blood aging, while others decreased, consistent with the transformation of endogenous metabolites (Figure 2d–e). As the raw data were not grouped, these features were subsequently manually curated to remove redundant signals (e.g., isotopes, adducts, and fragments) and low-quality peaks (e.g., poor signal-to-noise ratio or peak shape), resulting in a reduced set of 20 candidate biomarkers (10 per ionization mode), including spermidine, NAD^+^, glutamate, cystine, and several unknowns (Supporting Information S1).

To further interpret the observed temporal patterns, targeted degradation experiments were performed by using pure standards. These experiments enabled the tentative assignment of relationships intended to form ratios that remain independent of donor variability and sample deposition amount, rather than relying on relative metabolite concentrations. Spermidine formed a product with a higher *m*/*z* value of 184.1420 (Supplementary Figure S5a), potentially reflecting oxidation or adduct formation. The observed mass does not match that of a potassium adduct. NAD^+^ degraded into niacinamide (Supplementary Figure S5b), consistent with the cleavage of the nicotinamide moiety, and appeared to undergo further degradation over time.

An LC-MS feature increasing with time presented a phosphatidylcholine headgroup fragment, prompting further investigation into phospholipid degradation. Aging experiments with two unsaturated phosphatidylcholines revealed the formation of oxidized products, including putative epoxide species and lysophosphatidylcholine (Supplementary Figure S5c–h), consistent with autoxidation and hydrolytic cleavage of membrane lipids. This finding also aligns with the formation of epoxides of lipids in fingermarks over time.^20,21^ Similarly, Sun et al. reported an increase in lysophosphatidylcholines in bloodstains over a 10-day period.^22^

Although tryptophan was not selected by the model, it was examined due to its established relevance in forensic aging studies.^10,23^ Tryptophan was found to convert into kynurenine and subsequently into kynurenic acid, along with three unidentified products (Supplementary Figure S5i–m). This agrees with oxidative pathways previously described under light and oxygen exposure in aqueous solutions.^24^ The dry state of a bloodstain may further accelerate oxidation reactions because of its increased surface area. To our knowledge, however, these sequential transformations and associated products have not been previously described in the context of bloodstain aging.

Ergothioneine, also selected based on prior studies,^10,25^ formed both hercynine and a dimer (Supplementary Figure S5n–o). Notably, hercynine is a known degradation product of ergothioneine^26^ but has not previously been described as a bloodstain aging marker.

Additional metabolite-product relationships were identified for abundant metabolites found in bloodstains but not directly selected by the model, including BHB, palmitoyl carnitine, and glutathione species (Supplementary Figure S5p–r). BHB (C_4_H_8_O_3_) was observed to form a product consistent with that of C_4_H_6_O_4_, indicating extensive oxidation processes under ambient conditions. Similarly, carnitine was detected as a product of palmitoyl carnitine, consistent with hydrolytic cleavage of the ester bond, while reduced glutathione was observed alongside oxidized glutathione, reflecting redox processes. Conversely, a few model-selected metabolites, such as glutamate and cystine, did not exhibit measurable degradation products, which may reflect pathways not captured under experimental conditions.

Unfortunately, not all model-selected features could be fully assigned despite extensive efforts (Supplementary Figures S6–S7). MS/MS experiments were performed, and spectral matching was attempted using public databases as well as *in silico* annotation tools (SIRIUS), but without successful identification. This likely reflects that these features represent secondary degradation products formed during bloodstain aging, which are not well represented in current spectral libraries.

Taken together, the identified biomarkers represent different but interconnected aspects of blood aging, including oxidative damage, metabolic decline, and structural deterioration. A final panel of 51 biomarkers was selected for targeted analysis, comprising 20 biomarkers identified by the model, two phospholipids based on the observed phosphatidylcholine fragment, two biomarkers supported by previous literature (tryptophan and ergothioneine^10,23,25^ ), 17 experimentally confirmed degradation products, and a few additional high-intensity metabolites from the untargeted data set. This combined selection strategy ensures that the biomarker panel reflects not only an association with time but also the underlying chemical relevance. The full list can be found in Supplementary data S1 and Supplementary Table S2.

### Improving Biomarker Reliability Using Targeted LC-MS/MS Data to Predict Bloodstain Age

To develop prediction models, we generated a data set using our targeted semiquantitative LC-MS/MS analysis comprising 51 biomarkers. This data set included samples from the previously obtained untargeted data set and a new set of bloodstains with variable volumes collected from 53 donors and exposed to controlled environmental conditions (see Figure 1 and Supplementary Table S1). In total, 229 samples were used for model training. However, prior to modeling, all the biomarker intensities were transformed into pairwise ratios, including both metabolite-product pairs and unrelated pairs. Transforming the biomarkers into ratios acts as a form of “self-normalization”, intended to reduce variability related to sample amount, matrix effects, and other technical factors, while preserving biologically meaningful variation. This procedure yielded a total of 1275 ratios between all 51 biomarkers and provided better results than only including metabolite-product pairs as first intended.

Then, technical replicates were used to select ratios that showed both high biological variation and low technical variation (Supplementary Figure S8). Pairwise ratios with a Pearson *r* > 0.95 between all replicates were kept, leaving 91 stable ratios with approximately normally distributed values (Supplementary Figure S9). The ratio data show a strong pattern of time since deposition in the PCA score plot, with most of the variation (79%) present on the first two principal components (Figure 3a). This separation indicates that the self-normalization strategy enhances the temporal structure in the data while suppressing noninformative variation, supporting its utility for downstream predictive modeling.

**Figure 3:**
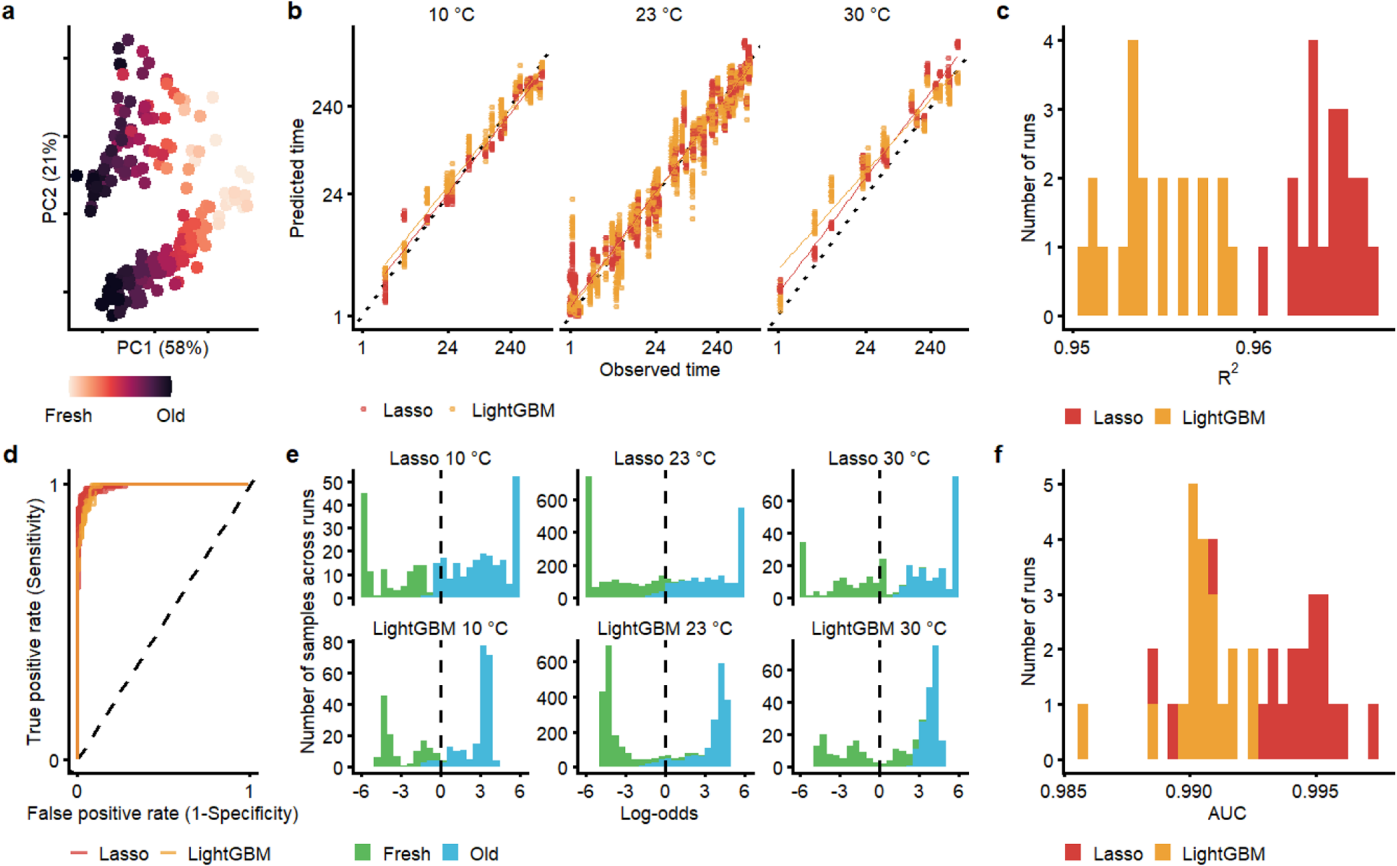
Time-since-deposition machine learning models of the targeted LC-MS/MS data. (a) PCA-scores plot of 229 bloodstains using stable biomarker ratios, with color indicating sample age. PC1 describes 58% of the total variance connected to time-since-deposition. (b) Regression results for bloodstains stored at different temperatures. (c) R^2^ values for independent machine learning runs with repeated cross-validations in the regression analysis. (d) Classification ROC curves for independent machine learning runs. (e) Classification results for samples stored at different temperatures when classifying samples as either fresh (<60 h) or old (>60 h). (f) AUC values for independent machine learning runs with repeated cross-validations.

Then we fitted two regression models, Lasso and LightGBM, to the stable ratio data. Time since deposition was modeled with “degree-hours” (temperature*hours) to better account for temperature effects, in contrast to using hours, which underpredicted at lower temperatures when trained on samples at room temperature (Wilcoxon rank-sum exact test, *p* < 2 × 10^–16^, Supplementary Figure S10). Additionally, we checked for possible effects of humidity and found moderate small effects when using Lasso, but substantial effects of high humidity (70%) when using LightGBM (Supplementary Figure S11). While the experimental conditions do not capture the full range of possible humidity variations, the results suggest that humidity has a measurable effect under more extreme conditions.

We found a strong correlation between observed and predicted time-since-deposition using both the Lasso and the LightGBM model for the entire studied temperature range of 10–30 °C (Figure 3b). Both models showed excellent overall performance (R^2^ > 0.95), with Lasso being superior across multiple independent machine learning runs (Figure 3c, Wilcoxon rank-sum exact test, *p* = 1.1 × 10^–11^). The same pattern was found for RMSE in different time windows, with Lasso having an RMSE of 6.6 h and LightGBM 13.6 h for samples stored <60 h. Prediction errors within this range are considered sufficiently small to be operationally useful to distinguish whether a bloodstain was deposited within the first 24–60 h. A threshold of 60 h was chosen in collaboration with police investigators, who deemed it relevant for severe criminal cases in Denmark, as they are often present within hours and can collect samples within the given time frame.

For samples stored longer, LightGBM predicted better than Lasso but with overall high RMSE values (76.6 and 69.9 h for samples stored up to 10 days, and 173.3 and 135.1 h for samples stored longer than 10 days). We did not observe any clear bias in prediction performance for 10 and 23 °C, but samples stored at 30 °C were generally predicted to be older, although the bias seemed to be smaller as time increased (Figure 3b, 30 °C). This time-dependent bias may reflect accelerated degradation kinetics at higher temperatures, particularly during the early stages of bloodstain aging. At longer time scales, however, degradation processes may approach saturation or converge toward similar end states, thereby reducing the relative impact of temperature differences on the metabolic profile.

Compared to previous metabolomics-based studies, which primarily report relative changes in metabolite levels rather than precise temporal predictions, the low RMSE values (<60 h) highlight the quantitative performance of the present approach, especially considering the inclusion of multiple donors and varying environmental conditions. Furthermore, previously reported studies have demonstrated substantial variability in metabolite degradation due to environmental factors,^12–14^ which likely also contributes to the increased prediction errors observed for longer time intervals in our study.

As a complementary strategy, we also tried to classify samples as either fresh (less than 60 h) or old (more than 60 h) using Lasso and LightGBM classification models to obtain a probability for each prediction. This allowed us to get a confidence estimate for each prediction, as probabilistic outputs (log-odds) can be directly interpreted in forensic decision-making. Most of the samples could be predicted during cross-validation with very high confidence (AUC > 0.98, Figure 3d) and with a slightly better performance for Lasso models (Figure 3f, Mann–Whitney, *p* = 2.9 × 10^–7^). Closer inspection revealed that LightGBM showed AUCs similar to those of Lasso but with lower certainty (closer to the decision boundary, Figure 3e). Samples that were misclassified were close to the 60-h threshold (Supplementary Figure S12), and all misclassifications showed log-odds close to 0 regardless of temperature (Figure 3e). The limitation of splitting the outcome label (age) into two classes (below or above 60 h) is that we lose resolution. However, our dual modeling strategy combines the interpretability of regression with the operational utility of classification, addressing investigative needs, such as threshold-based decisions.

Importance analysis revealed that only 33 ratios had an importance score greater than 5 in at least one model after scaling the importances to a maximum of 100 for each model and objective (Supplementary Figure S13). Single molecules were included in several highly important ratios, e.g., tryptophan/X, which indicates possible high covariance. The correlation heatmap (Supplementary Figure S14) shows three clusters of tightly correlated ratios, which means that all ratios within a cluster will have very similar signals, and both Lasso and LightGBM will not show a strong preference for any of them. Two of the clusters show a strong between-cluster negative correlation, likely indicating ratios decreasing and increasing with time since deposition.

Together, these findings indicate that the number of biomarkers can be reduced without substantial loss of information, as clusters of correlated ratios effectively capture similar underlying processes. Additionally, we initially hypothesized that metabolite-product pairs could offer a normalization-free approach for age estimation. However, these pairs do not seem to be part of the most important ratios in the models. Thus, this approach did not perform as intended, possibly because several metabolites degrade into multiple products rather than a single, well-defined counterpart.

Despite this, the broader ratio-based strategy proved effective. The use of ratios seems to mitigate interindividual variability in baseline metabolite levels. Absolute concentrations of metabolites are known to vary substantially between individuals due to factors such as age, sex, and health status.^27–31^ For example, the concentration of ergothioneine has previously been shown to increase significantly with donor age.^25^ Consequently, reliance on the absolute abundances of such metabolites could confound bloodstain age estimation. Ratio-based markers address this limitation by scaling each biomarker relative to another, thereby reducing sensitivity to interindividual baseline differences and improving robustness across donors. This interpretation is supported by PCA clustering and repeated cross-validation, which contributed to the good model performance of both regression and classification models.

### Validation of Prediction Performance

To further validate our Lasso model, we analyzed two independent data sets created under different conditions (validation sets 1 and 2). When predicting time since deposition, the first set (kept at controlled settings) validated nicely with an R^2^ of 0.83 and a classification performance of 0.97 (AUC, Figure 4). Validation set 2 (kept at various uncontrolled settings) showed more variable performance (R^2^ = 0.30), primarily due to a set of samples being placed on a west-facing windowsill where they experienced cooling effects from the outside environment and variable solar exposure—conditions that fall outside of our training data distribution. Notably, even under these challenging conditions, the classification model maintained a reasonable performance (AUC = 0.85).

**Figure 4:**
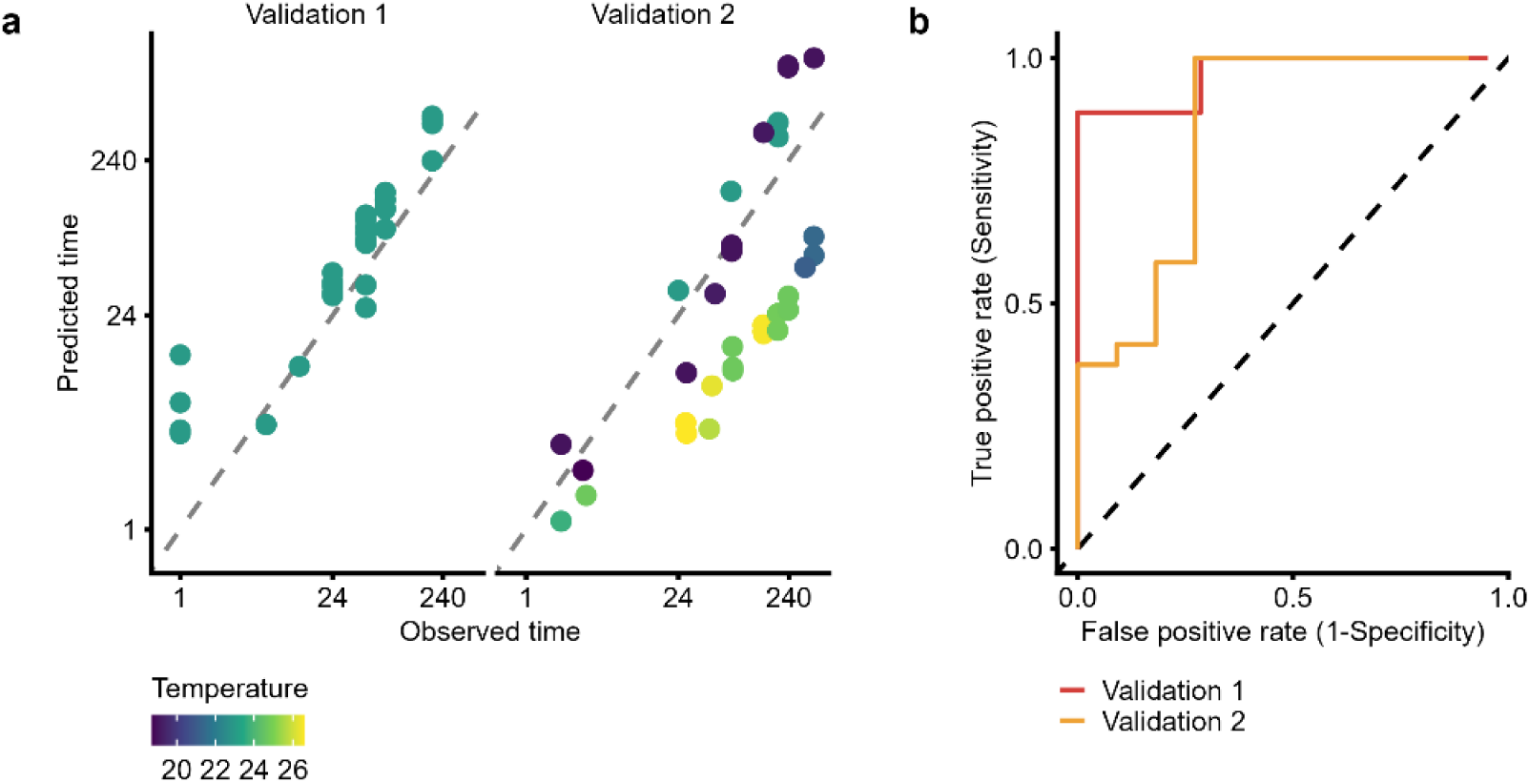
Time-since-deposition prediction of two independent validation data sets. (a) Predicted time-since-deposition on the two independent validation sets. (b) ROC curves for the classification of the samples as either <60 h or >60 h for the two validation sets.

This reduced accuracy under uncontrolled conditions further highlights the complexity of environmental interactions on blood aging. In particular, the affected samples were exposed to fluctuating cold temperatures and sampled on a sunny day, resulting in artificially high temperature readings. Although modeling time as degree-hours substantially improved temperature handling across the 10–30 °C range, this approach seems most reliable under relatively stable conditions. As with any forensic method, the current validation defines specific operating conditions. Thus, the method has been validated for blood deposited indoors on smooth substrates under stable temperature and humidity, which are conditions that represent a substantial proportion of indoor crime scenes. Conditions outside this range, such as prolonged UV exposure, very high humidity, or blood pools rather than stains, warrant further study to establish their impact on prediction accuracy.

## Conclusion

Estimating the time since the deposition of bloodstains remains a longstanding challenge in forensic science. Although numerous studies have documented biochemical changes over time, these findings have, to our knowledge, not been translated into operationally viable methods. Our study addresses this gap by demonstrating that machine learning models trained on metabolic blood profiles can predict bloodstain age across multiple individuals, eliminating donor-specific bias under defined environmental conditions. Central to this achievement is a targeted semiquantitative LC-MS/MS method based on selected biomarkers and the use of ratios for self-normalization. Importantly, the method achieves high predictive performance within forensically relevant time scales, with low prediction errors for early time points and near-perfect classification of recent (<60 h) versus older bloodstains.

A key strength of the approach lies in the external validation of predictive models on fully independent data sets, confirming robustness under typical indoor conditions and underscoring the method’s reliability for common forensic scenarios. The ability to provide both continuous time estimates and probabilistic classification outputs further enables forensic practitioners to communicate results and uncertainty for casework.

In relation to other bloodstain age techniques, such as rapid on-site spectroscopic- or imaging-based screening methods, LC-MS/MS-based analysis may serve as a confirmatory approach to improve confidence, accuracy, and evidential value. This is analogous to other multistep validation strategies commonly employed in forensic practice.

While the current study demonstrates strong performance under controlled indoor conditions, limitations remain. Prediction accuracy decreases for longer time intervals and under highly variable environmental conditions, highlighting the complexity of long-term degradation processes and the influence of external factors that are not fully captured by the current models. Further work is therefore needed to expand validation across a broader range of substrates and environmental conditions. Our ongoing efforts also include optimization of sample handling procedures, including transport and storage after collection, as well as testing on authentic forensic samples. In addition, future work will focus on further investigation of the most informative biomarker ratios, as the current models indicate that certain biomarkers contribute more strongly than others, while some appear to have a limited impact on predictive performance.

Overall, this work bridges the gap between biochemical observations and operational forensic tools by providing an interpretable framework for bloodstain age estimation across individuals. With further validation and development, the approach has the potential to become a valuable tool for forensic timeline reconstruction and decision-making in criminal investigations.

## Supplementary Material









## Data Availability

The raw data are publicly available at MetaboLights with the study identifier MTBLS14094: https://www.ebi.ac.uk/metabolights/MTBLS14094.

## Notes

Data S1 includes feature selection information from the untargeted data set (XLSX)
Source data and data analysis code for the untargeted data analysis (ZIP)
Figure S1. Contributors of variance in the untargeted negative and positive ESI data. Figure S2. Normalized feature intensities before filtering in positive ESI data. Figure S3. Normalized feature intensities before filtering in negative ESI data. Figure S4. Filtering process for negative ESI data (same as Figure 2a–c in the main text). Figure S5. Identification and annotation of breakdown products from standard compounds. Figure S6. High-resolution MS/MS spectra of unknown breakdown products in positive ESI mode. Figure S7. High-resolution MS/MS spectra of unknown breakdown products in negative ESI mode. Figure S8. Filtering of ratios using technical replicates. Figure S9. Distribution of log2­(ratio) values in the targeted training data. Figure S10. Effect of temperature and using either hours or degree-hours as response. Figure S11. Effect of humidity. Figure S12. Cross-validated log-odds for different time points across 20 independent runs. Figure S13. Ratio importance measures from different models and objectives (Lasso, LightGBM and regression, and classification). Figure S14. Correlation heatmap using Spearman rank correlation of the 91 ratios, showing large clusters of highly correlated ratios. Table S1. Donor characteristics, sampling practices, and bloodstain aging conditions for the different data sets. Table S2. List of components for the targeted LC-MS/MS method, including MRM transitions and retention times. (PDF)
Source data and data analysis code for the targeted data analysis and independent validation (ZIP)

## Abbreviations

(PC­(16:0/18:1)): 1-palmitoyl-2-oleoyl-*sn*-glycero-3-phosphocholine
PC­(18:0/18:2): 1-stearoyl-2-linoleoyl-*sn*-glycero-3-phosphocholine
BHB: 3-hydroxybutyric acid
MeCN: acetonitrile
NAD^+^: β-nicotinamide adenine dinucleotide
DDA: data-dependent acquisition
ESI: electrospray ionization
LC-MS: liquid chromatography–mass spectrometry
MeOH: methanol
GSSG: oxidized glutathione
QC: quality control
GSH: reduced glutathione
