# Supplementary material for: Donor-Independent Metabolomics Enables Bloodstain Age Determination at Crime Scenes
Source: J Proteome Res. 2026 Jun 19;25(7):3671–80. doi: 10.1021/acs.jproteome.6c00199 (PMC13339758; doi:10.1021/acs.jproteome.6c00199)
Supplement: Supplementary file 3 [file pr6c00199_si_003.pdf]

Supplementary Information

Donor-independent metabolomics enables bloodstain age determination at crime scenes

Kirstine L. Nielsen[1*], Johan K. Lassen[1,2], Ida Marie M. Løber[1], Frederik Skovbo[1], Kim Frisch[1], Tomasz P. Czaja[3], Bekzod Khakimov[3], Søren B. Engelsen[3], Mogens Johannsen[1], Palle Villesen[2,4*]

*Corresponding authors. Email: klyn@forens.au.dk, palle@birc.au.dk

Table of Contents

Figure S1. Contributors of variance in the untargeted negative and positive ESI data.
Figure S2. Normalized feature intensities before filtering in positive ESI data.
Figure S3. Normalized feature intensities before filtering in negative ESI data.
Figure S4. Filtering process for negative ESI data (same as Fig. 2a-c in the main text).
Figure S5. Identification and annotation of breakdown products from standard compounds.
Figure S6. High resolution MS/MS spectra of unknown breakdown products in positive ESI mode.
Figure S7. High resolution MS/MS spectra of unknown breakdown products in negative ESI mode.
Figure S8. Filtering of ratios using technical replicates.
Figure S9. Distribution of log2(ratio) values in the targeted training data.
Figure S10. Effect of temperature and using either hours or degreehours as response.
Figure S11. Effect of humidity.
Figure S12. Cross-validated Log-odds for different timepoints for 20 independent runs.
Figure S13. Ratio importance measures from different models and objectives (Lasso and LightGBM and regression and classification.
Figure S14. Correlation heatmap using spearman rank correlation of the 91 ratios showing large clusters of highly correlated ratios.
Table S1. Donor characteristics, sampling practice, and bloodstain aging conditions for the different datasets.
Table S2. List of components for the targeted LC-MS/MS method including MRM transitions and retention times.
Data S1: Includes feature selection information from the untargeted dataset
untargeted_analysis.zip: Source data and data analysis code for the untargeted data analysis
targeted_analysis_zip: Source data and data analysis code for the targeted data analysis and independent validation

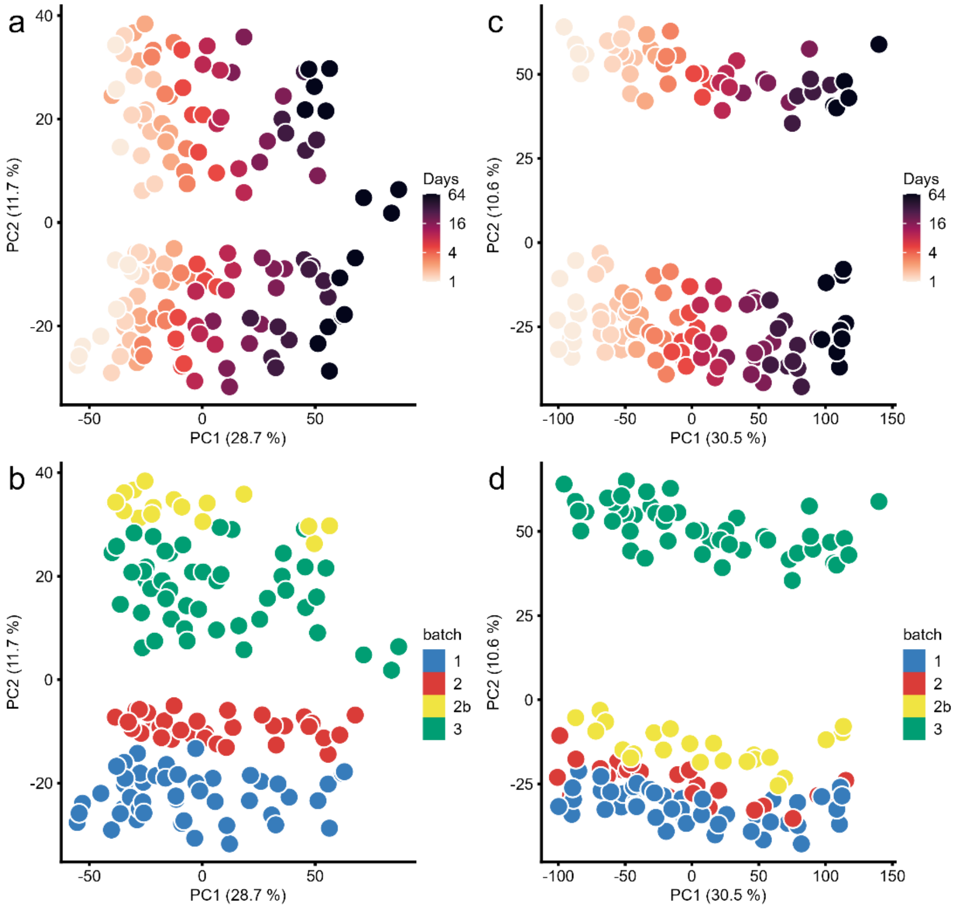

Figure S1. Contributors of variance in the untargeted (a, b) negative and (c, d) positive ESI data.

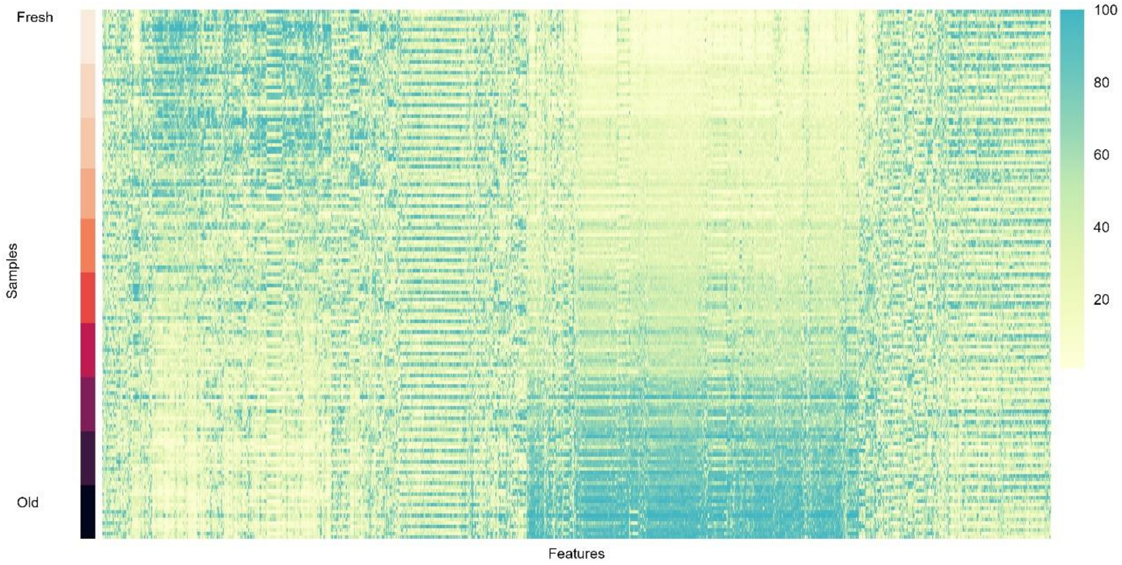

Figure S2. Normalized feature intensities before filtering in positive ESI data.

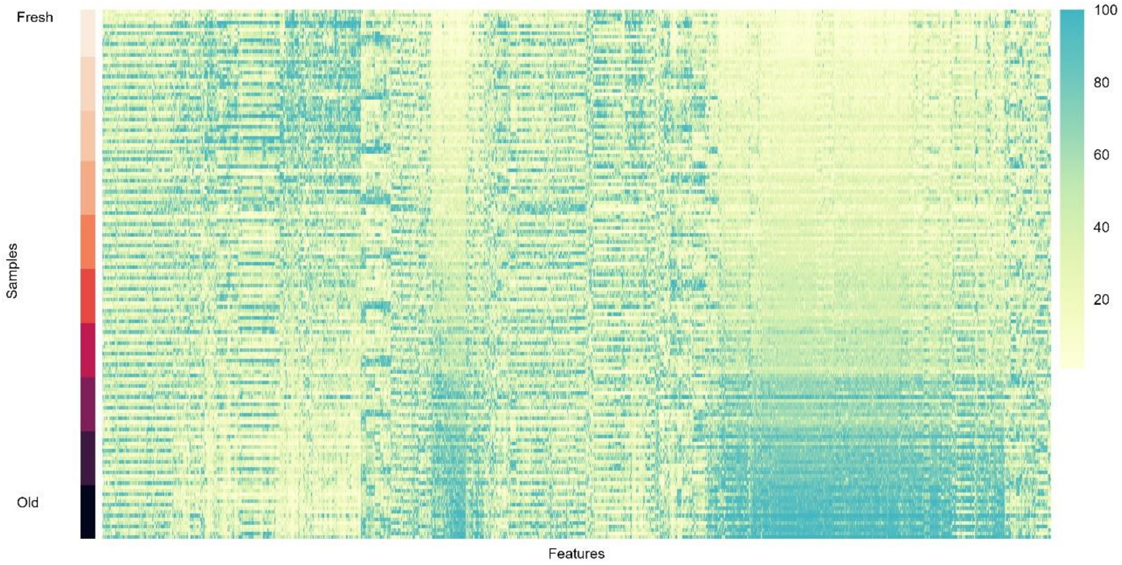

Figure S3. Normalized feature intensities before filtering in negative ESI data.

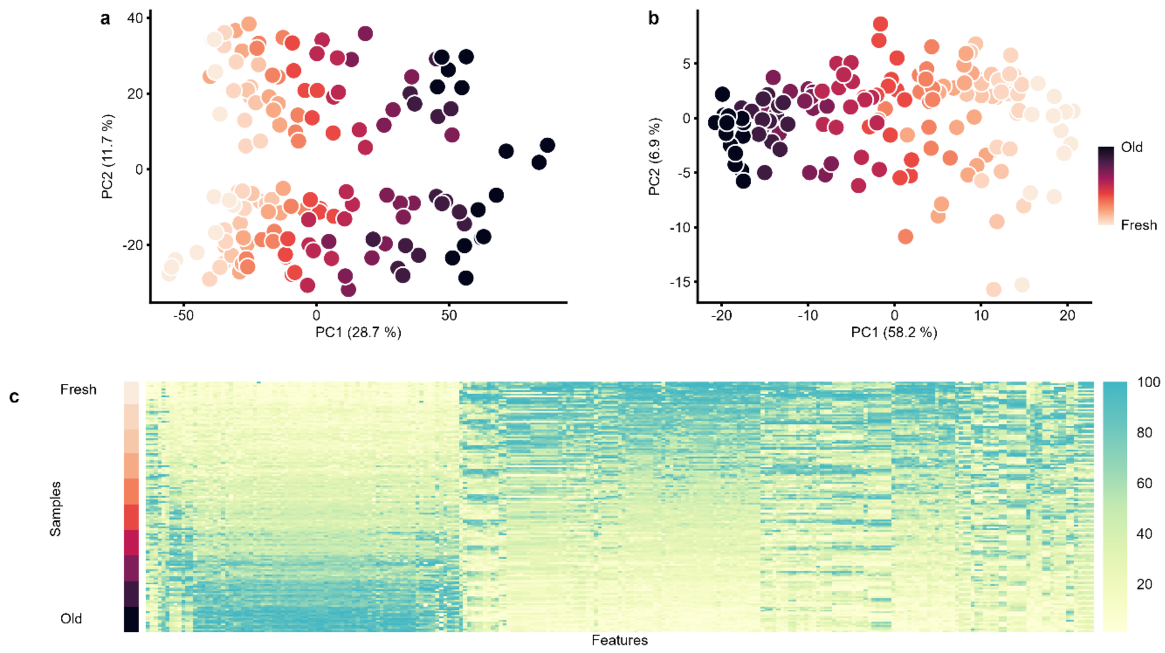

Figure S4. Filtering process for negative ESI data (same as Fig. 2a-c in the main text).

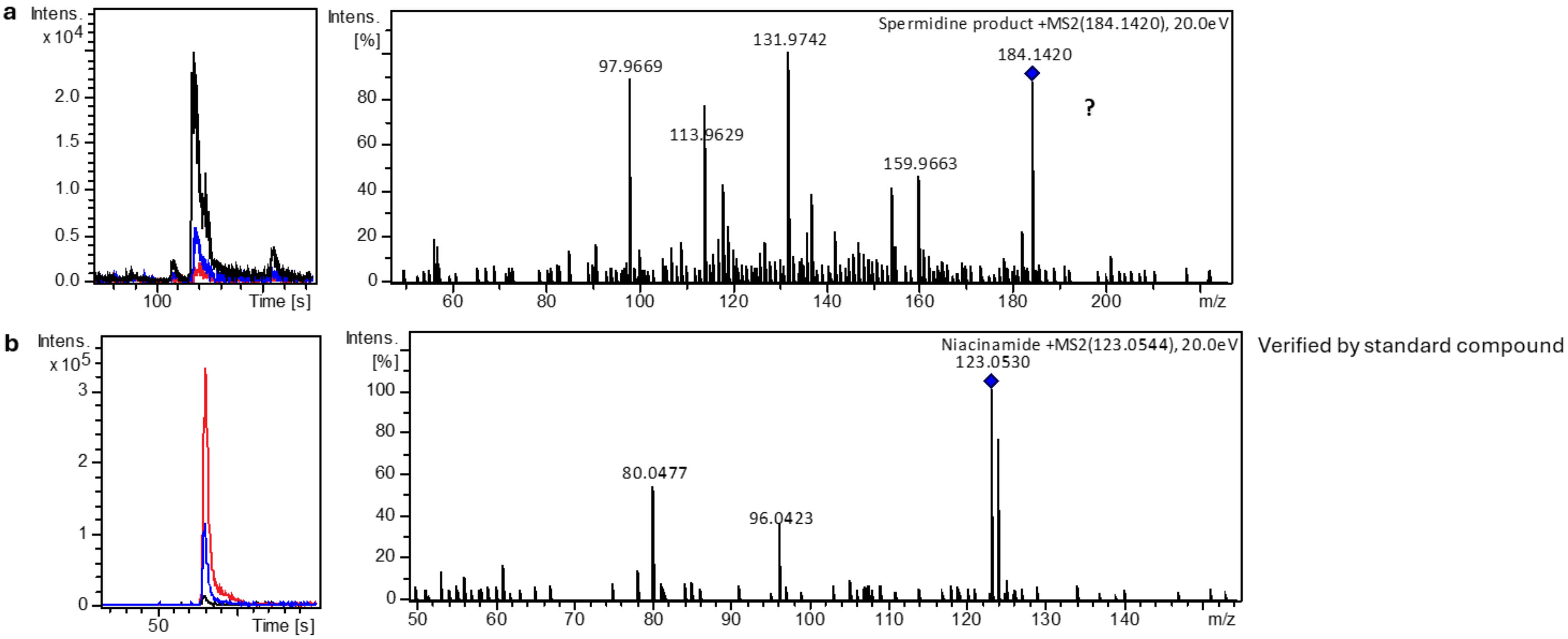

Figure S5. Identification and annotation of breakdown products from standard compounds. Extracted ion chromatograms of 1-day old (red), 7-day old (blue), and 14-day old (black) samples including high-resolution ms/ms spectra with possible structural suggestions of breakdown products. The precursor ions are marked with a blue pane. **(a)** Spermidine product. **(b)** Niacinamide peak found in aged β-Nicotinamide adenine dinucleotide (NAD+) samples. **(c-e)** PC(34:1) product 1, 2, and 3 found in aged samples of 1-palmitoyl-2-oleoyl-sn-glycero-3-phosphocholine (PC(16:0/18:1)). **(f-h)** PC(36:1) product 1, 2, and 3 found in aged samples of 1-palmitoyl-2-linoleoyl-sn-glycero-3-phosphocholine (PC(16:0/18:2)). **(i)** Kynurenine peak found in aged tryptophan samples. **(j-l)** Kynurenine product 1, 2, and kynurenic acid found in aged kynurenine samples, respectively. **(m)** Kynurenic acid product. **(n-o)** Ergothioneine product 1 (identified as hercynine) and 2 (ergothioneine dimer). **(p)** 3-hydroxybutyric acid (BHB) product. **(q)** Carnitine peak found in aged palmitoyl carnitine samples. **(r)** Reduced glutathione in aged glutathione (oxidized) samples.

c

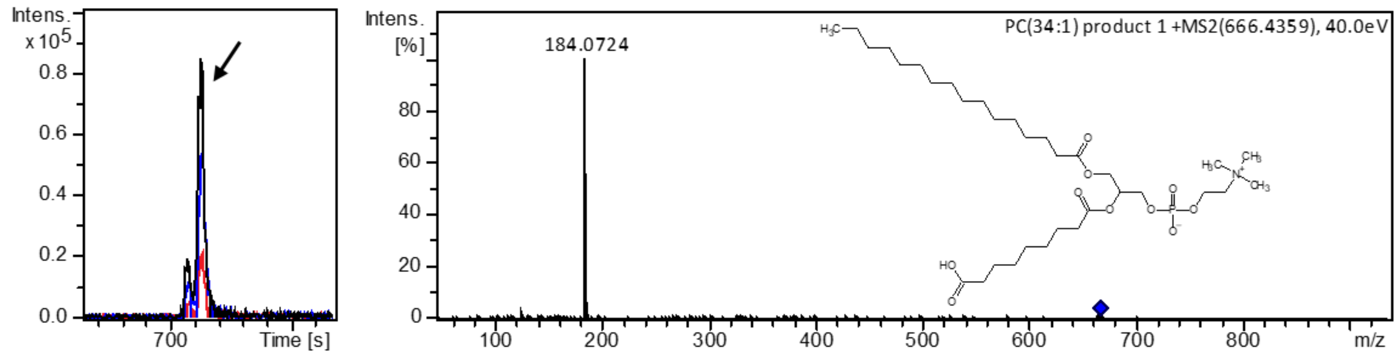

mass	formula	Error (ppm)
666.4359	C33H64NO10P	2.7
184.0724	C5H15NO4P	4.9

d

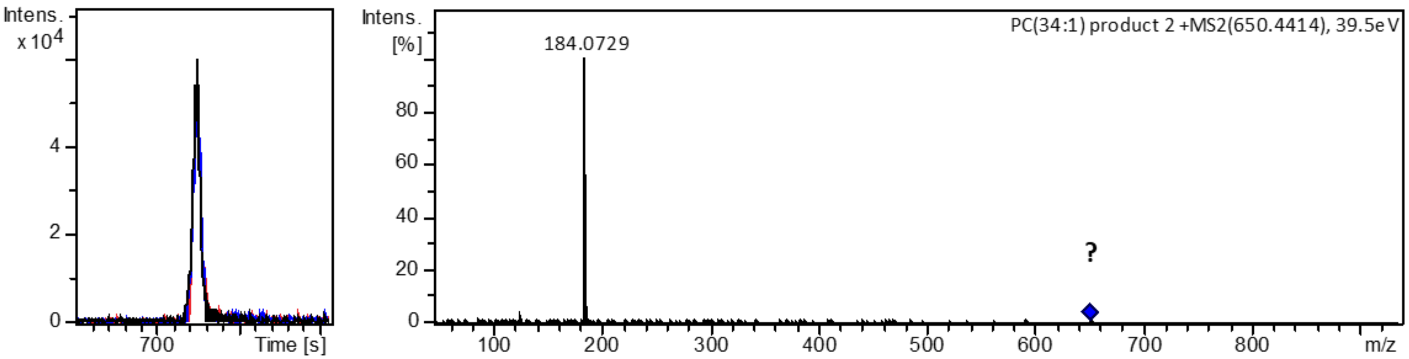

mass	formula	Error (ppm)
650.4414	?	
184.0729	C5H15NO4P	2.2

Figure S5 (continued). Identification and annotation of breakdown products from standard compounds. Extracted ion chromatograms of 1-day old (red), 7-day old (blue), and 14-day old (black) samples including high-resolution ms/ms spectra with possible structural suggestions of breakdown products. The precursor ions are marked with a blue pane. (**a**) Spermidine product. (**b**) Niacinamide peak found in aged β-Nicotinamide adenine dinucleotide (NAD+) samples. (**c-e**) PC(34:1) product 1, 2, and 3 found in aged samples of 1-palmitoyl-2-oleoyl-sn-glycero-3-phosphocholine (PC(16:0/18:1)). (**f-h**) PC(36:1) product 1, 2, and 3 found in aged samples of 1-palmitoyl-2-linoleoyl-sn-glycero-3-phosphocholine (PC(16:0/18:2)). (**i**) Kynurenine peak found in aged tryptophan samples. (**j-l**) Kynurenine product 1, 2, and kynurenic acid found in aged kynurenine samples, respectively. (**m**) Kynurenic acid product. (**n-o**) Ergothioneine product 1 (identified as hercynine) and 2 (ergothioneine dimer). (**p**) 3-hydroxybutyric acid (BHB) product. (**q**) Carnitine peak found in aged palmitoyl carnitine samples. (**r**) Reduced glutathione in aged glutathione (oxidized) samples.

e

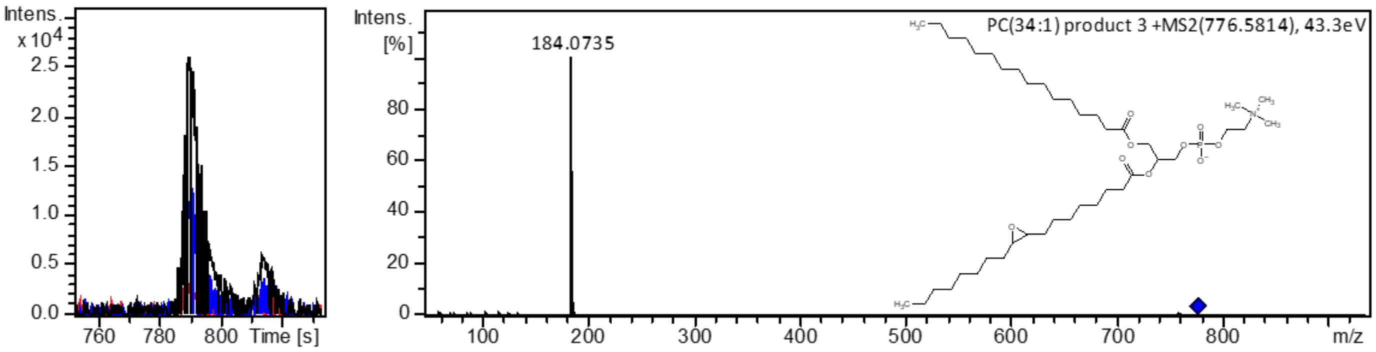

mass	formula	Error (ppm)
776.5814	C42H82NO9P	1.8
184.0735	C5H15NO4P	1.1

f

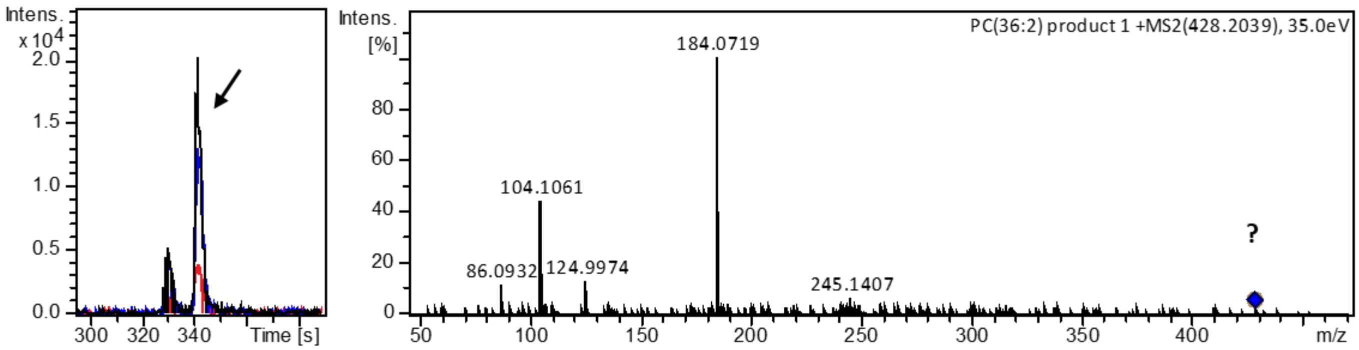

Mass	formula	Error (ppm)
184.0719	C5H15NO4P	7.6
124.9974	C2H6O4P	19.2
104.1061	C5H14NO	8.6
86.0932	C5H12N	37.2

Figure S5 (continued). Identification and annotation of breakdown products from standard compounds. Extracted ion chromatograms of 1-day old (red), 7-day old (blue), and 14-day old (black) samples including high-resolution ms/ms spectra with possible structural suggestions of breakdown products. The precursor ions are marked with a blue pane. (**a**) Spermidine product. (**b**) Niacinamide peak found in aged β-Nicotinamide adenine dinucleotide (NAD+) samples. (**c-e**) PC(34:1) product 1, 2, and 3 found in aged samples of 1-palmitoyl-2-oleoyl-sn-glycero-3-phosphocholine (PC(16:0/18:1)). (**f-h**) PC(36:1) product 1, 2, and 3 found in aged samples of 1-palmitoyl-2-linoleoyl-sn-glycero-3-phosphocholine (PC(16:0/18:2)). (**i**) Kynurenine peak found in aged tryptophan samples. (**j-l**) Kynurenine product 1, 2, and kynurenic acid found in aged kynurenine samples, respectively. (**m**) Kynurenic acid product. (**n-o**) Ergothioneine product 1 (identified as hercynine) and 2 (ergothioneine dimer). (**p**) 3-hydroxybutyric acid (BHB) product. (**q**) Carnitine peak found in aged palmitoyl carnitine samples. (**r**) Reduced glutathione in aged glutathione (oxidized) samples.

g

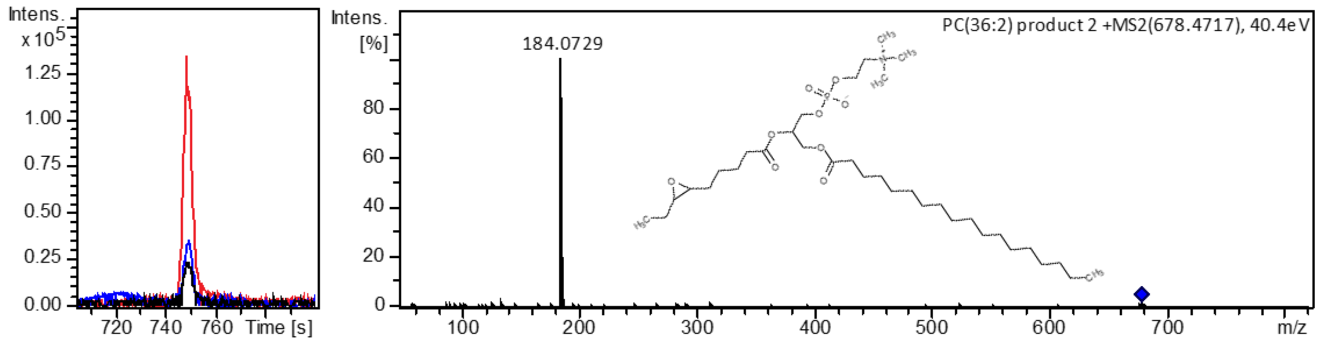

mass	formula	Error (ppm)
678.4717	C35H68NO9P	1.9
184.0729	C5H15NO4P	2.2

h

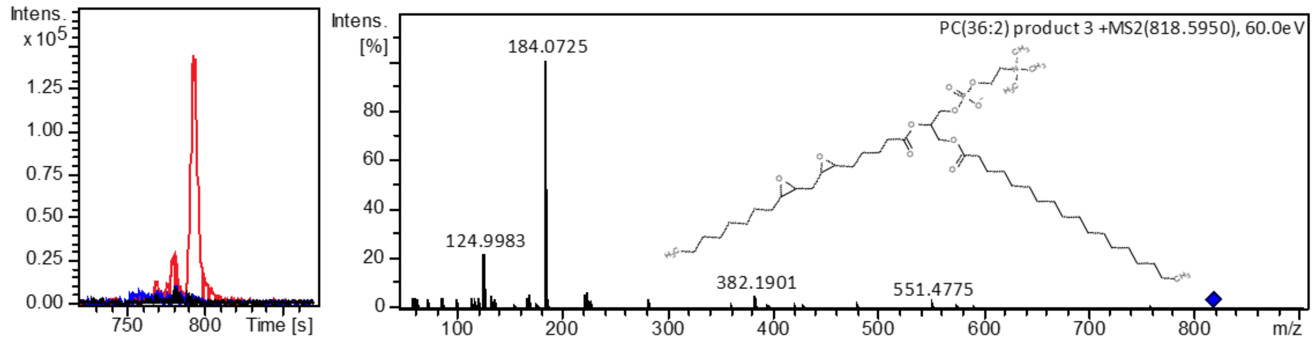

mass	formula	Error (ppm)
818.5950	C44H84NO10P	5.4
551.4775	?	
382.1901	C16H33NO7P	23.0
184.0729	C5H15NO4P	2.2
124.9983	C2H6O4P	12.0

Figure S5 (continued). Identification and annotation of breakdown products from standard compounds. Extracted ion chromatograms of 1-day old (red), 7-day old (blue), and 14-day old (black) samples including high-resolution ms/ms spectra with possible structural suggestions of breakdown products. The precursor ions are marked with a blue pane. **(a)** Spermidine product. **(b)** Niacinamide peak found in aged β-Nicotinamide adenine dinucleotide (NAD+) samples. **(c-e)** PC(34:1) product 1, 2, and 3 found in aged samples of 1-palmitoyl-2-oleoyl-sn-glycero-3-phosphocholine (PC(16:0/18:1)). **(f-h)** PC(36:1) product 1, 2, and 3 found in aged samples of 1-palmitoyl-2-linoleoyl-sn-glycero-3-phosphocholine (PC(16:0/18:2)). **(i)** Kynurenine peak found in aged tryptophan samples. **(j-l)** Kynurenine product 1, 2, and kynurenic acid found in aged kynurenine samples, respectively. **(m)** Kynurenic acid product. **(n-o)** Ergothioneine product 1 (identified as hercynine) and 2 (ergothioneine dimer). **(p)** 3-hydroxybutyric acid (BHB) product. **(q)** Carnitine peak found in aged palmitoyl carnitine samples. **(r)** Reduced glutathione in aged glutathione (oxidized) samples.

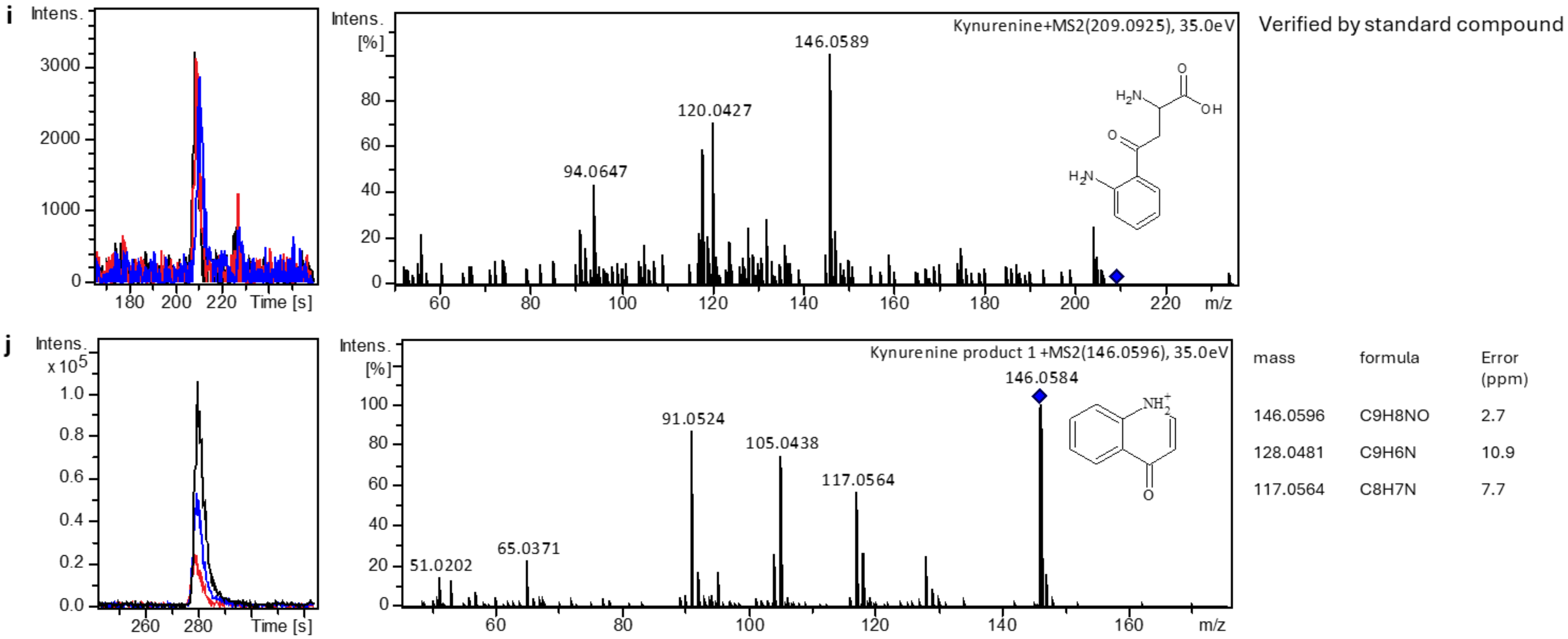

Figure S5 (continued). Identification and annotation of breakdown products from standard compounds. Extracted ion chromatograms of 1-day old (red), 7-day old (blue), and 14-day old (black) samples including high-resolution ms/ms spectra with possible structural suggestions of breakdown products. The precursor ions are marked with a blue pane. **(a)** Spermidine product. **(b)** Niacinamide peak found in aged β-Nicotinamide adenine dinucleotide (NAD+) samples. **(c-e)** PC(34:1) product 1, 2, and 3 found in aged samples of 1-palmitoyl-2-oleoyl-sn-glycero-3-phosphocholine (PC(16:0/18:1)). **(f-h)** PC(36:1) product 1, 2, and 3 found in aged samples of 1-palmitoyl-2-linoleoyl-sn-glycero-3-phosphocholine (PC(16:0/18:2)). **(i)** Kynurenine peak found in aged tryptophan samples. **(j-l)** Kynurenine product 1, 2, and kynurenic acid found in aged kynurenine samples, respectively. **(m)** Kynurenic acid product. **(n-o)** Ergothioneine product 1 (identified as hercynine) and 2 (ergothioneine dimer). **(p)** 3-hydroxybutyric acid (BHB) product. **(q)** Carnitine peak found in aged palmitoyl carnitine samples. **(r)** Reduced glutathione in aged glutathione (oxidized) samples.

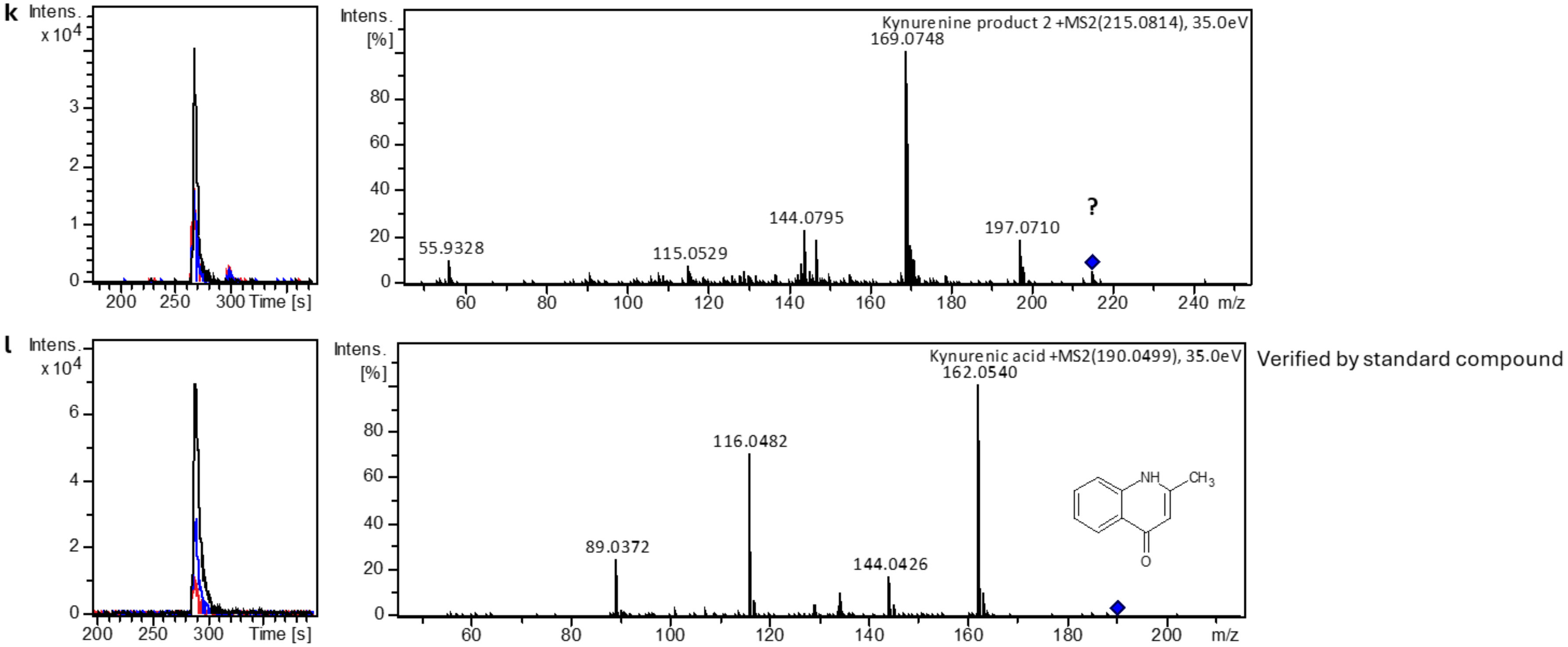

Figure S5 (continued). Identification and annotation of breakdown products from standard compounds. Extracted ion chromatograms of 1-day old (red), 7-day old (blue), and 14-day old (black) samples including high-resolution ms/ms spectra with possible structural suggestions of breakdown products. The precursor ions are marked with a blue pane. (**a)** Spermidine product. (**b)** Niacinamide peak found in aged β-Nicotinamide adenine dinucleotide (NAD+) samples. (**c-e)** PC(34:1) product 1, 2, and 3 found in aged samples of 1-palmitoyl-2-oleoyl-sn-glycero-3-phosphocholine (PC(16:0/18:1)). (**f-h)** PC(36:1) product 1, 2, and 3 found in aged samples of 1-palmitoyl-2-linoleoyl-sn-glycero-3-phosphocholine (PC(16:0/18:2)). (**i)** Kynurenine peak found in aged tryptophan samples. (**j-l)** Kynurenine product 1, 2, and kynurenic acid found in aged kynurenine samples, respectively. (**m)** Kynurenic acid product. (**n-o)** Ergothioneine product 1 (identified as hercynine) and 2 (ergothioneine dimer). (**p)** 3-hydroxybutyric acid (BHB) product. (**q)** Carnitine peak found in aged palmitoyl carnitine samples. (**r)** Reduced glutathione in aged glutathione (oxidized) samples.

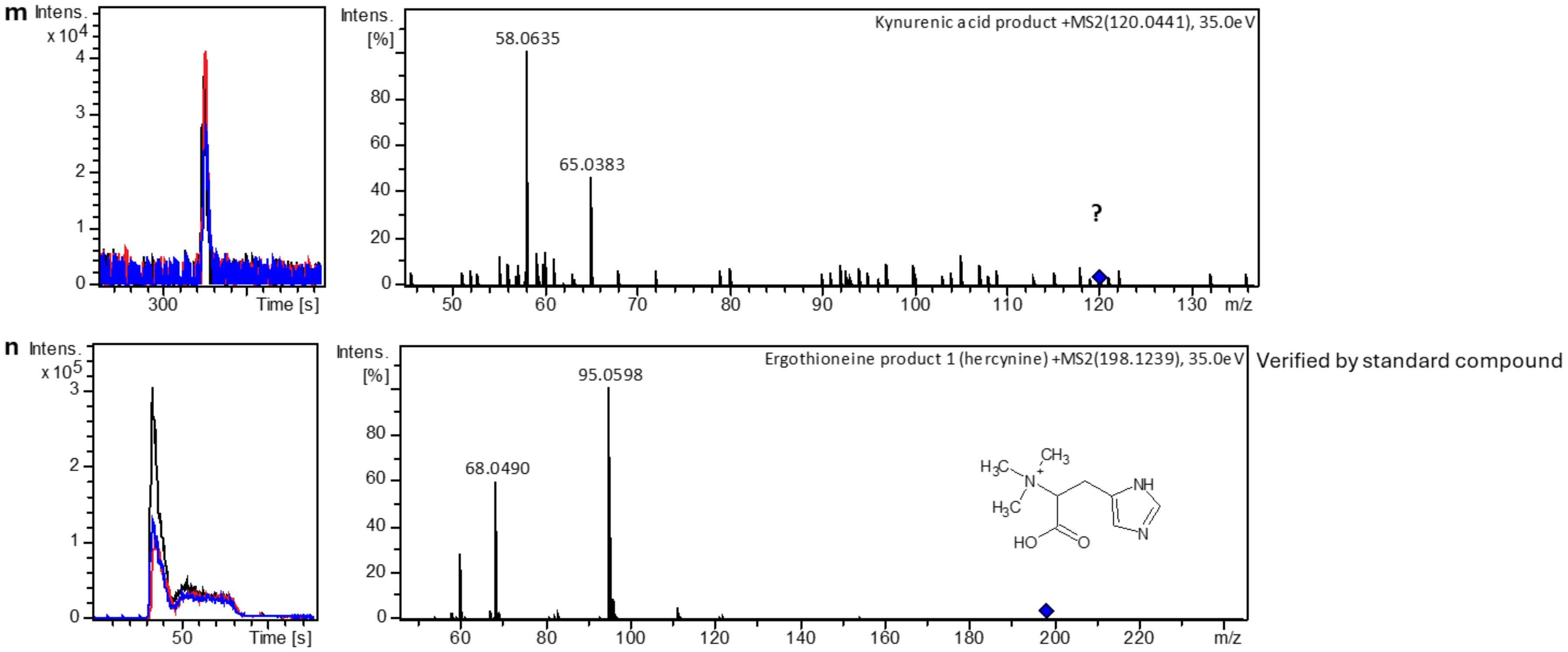

Figure S5 (continued). Identification and annotation of breakdown products from standard compounds. Extracted ion chromatograms of 1-day old (red), 7-day old (blue), and 14-day old (black) samples including high-resolution ms/ms spectra with possible structural suggestions of breakdown products. The precursor ions are marked with a blue pane. (**a**) Spermidine product. (**b**) Niacinamide peak found in aged β-Nicotinamide adenine dinucleotide (NAD+) samples. (**c-e**) PC(34:1) product 1, 2, and 3 found in aged samples of 1-palmitoyl-2-oleoyl-sn-glycero-3-phosphocholine (PC(16:0/18:1)). (**f-h**) PC(36:1) product 1, 2, and 3 found in aged samples of 1-palmitoyl-2-linoleoyl-sn-glycero-3-phosphocholine (PC(16:0/18:2)). (**i**) Kynurenine peak found in aged tryptophan samples. (**j-l**) Kynurenine product 1, 2, and kynurenic acid found in aged kynurenine samples, respectively. (**m**) Kynurenic acid product. (**n-o**) Ergothioneine product 1 (identified as hercynine) and 2 (ergothioneine dimer). (**p**) 3-hydroxybutyric acid (BHB) product. (**q**) Carnitine peak found in aged palmitoyl carnitine samples. (**r**) Reduced glutathione in aged glutathione (oxidized) samples.

o

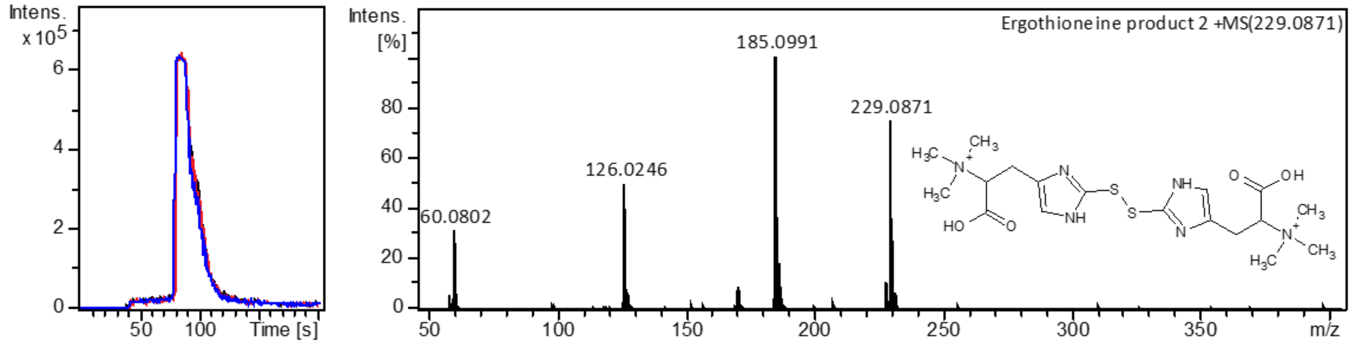

mass	formula	Error (ppm)
229.0871	C18H30N6O4S2(2+)	3.5
185.0991	C16H30N6S2(2+)	5.4
170.0746	C13H18N5O2S2(2+)	0.0
60.0802	C3H10N	10.0

p

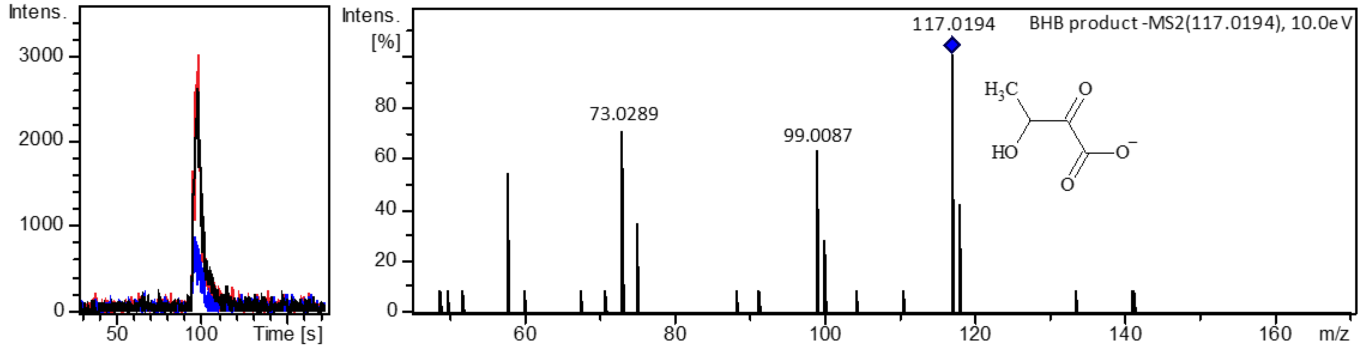

mass	formula	Error (ppm)
117.0194	C4H6O4	0.9
99.0087	C4H3O3	1.0
73.0289	C3H5O2	8.2

Figure S5 (continued). Identification and annotation of breakdown products from standard compounds. Extracted ion chromatograms of 1-day old (red), 7-day old (blue), and 14-day old (black) samples including high-resolution ms/ms spectra with possible structural suggestions of breakdown products. The precursor ions are marked with a blue pane. **(a)** Spermidine product. **(b)** Niacinamide peak found in aged β-Nicotinamide adenine dinucleotide (NAD+) samples. **(c-e)** PC(34:1) product 1, 2, and 3 found in aged samples of 1-palmitoyl-2-oleoyl-sn-glycero-3-phosphocholine (PC(16:0/18:1)). **(f-h)** PC(36:1) product 1, 2, and 3 found in aged samples of 1-palmitoyl-2-linoleoyl-sn-glycero-3-phosphocholine (PC(16:0/18:2)). **(i)** Kynurenine peak found in aged tryptophan samples. **(j-l)** Kynurenine product 1, 2, and kynurenic acid found in aged kynurenine samples, respectively. **(m)** Kynurenic acid product. **(n-o)** Ergothioneine product 1 (identified as hercynine) and 2 (ergothioneine dimer). **(p)** 3-hydroxybutyric acid (BHB) product. **(q)** Carnitine peak found in aged palmitoyl carnitine samples. **(r)** Reduced glutathione in aged glutathione (oxidized) samples.

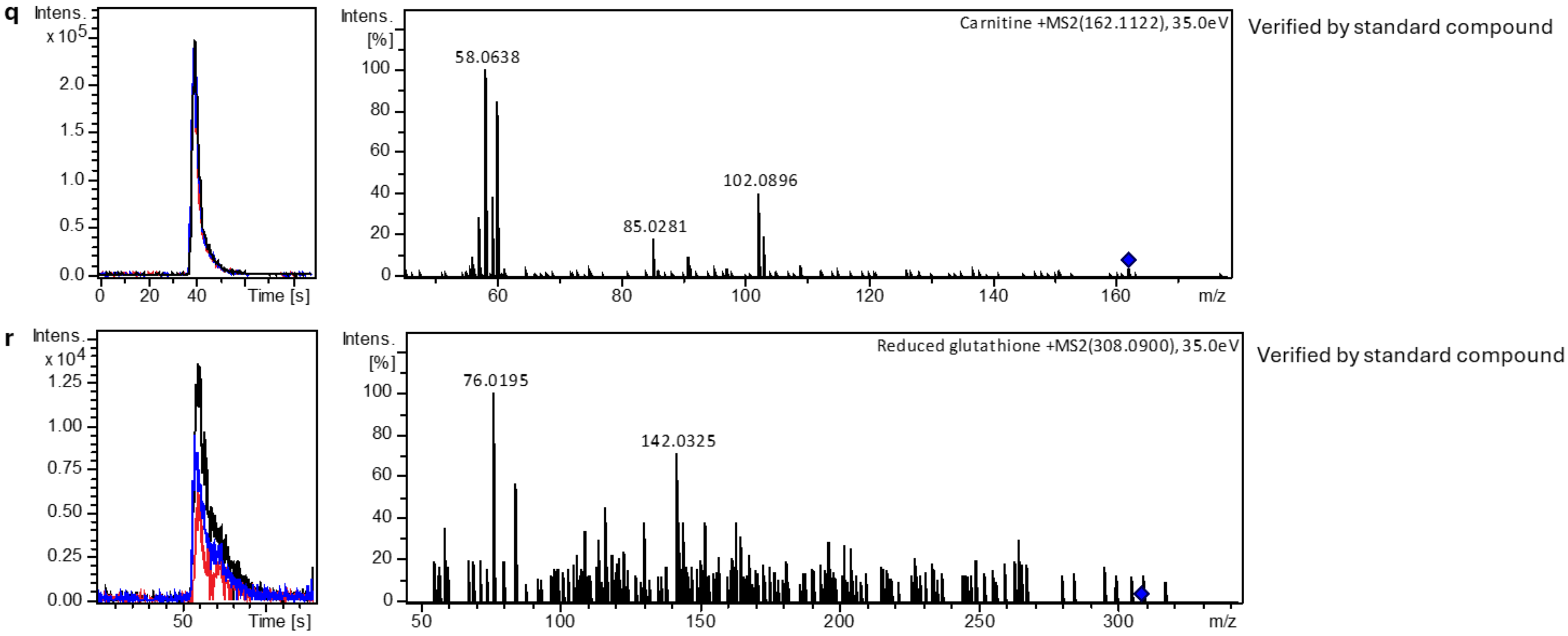

Figure S5 (continued). Identification and annotation of breakdown products from standard compounds. Extracted ion chromatograms of 1-day old (red), 7-day old (blue), and 14-day old (black) samples including high-resolution ms/ms spectra with possible structural suggestions of breakdown products. The precursor ions are marked with a blue pane. (**a**) Spermidine product. (**b**) Niacinamide peak found in aged β-Nicotinamide adenine dinucleotide (NAD+) samples. (**c-e**) PC(34:1) product 1, 2, and 3 found in aged samples of 1-palmitoyl-2-oleoyl-sn-glycero-3-phosphocholine (PC(16:0/18:1)). (**f-h**) PC(36:1) product 1, 2, and 3 found in aged samples of 1-palmitoyl-2-linoleoyl-sn-glycero-3-phosphocholine (PC(16:0/18:2)). (**i**) Kynurenine peak found in aged tryptophan samples. (**j-l**) Kynurenine product 1, 2, and kynurenic acid found in aged kynurenine samples, respectively. (**m**) Kynurenic acid product. (**n-o**) Ergothioneine product 1 (identified as hercynine) and 2 (ergothioneine dimer). (**p**) 3-hydroxybutyric acid (BHB) product. (**q**) Carnitine peak found in aged palmitoyl carnitine samples. (**r**) Reduced glutathione in aged glutathione (oxidized) samples.

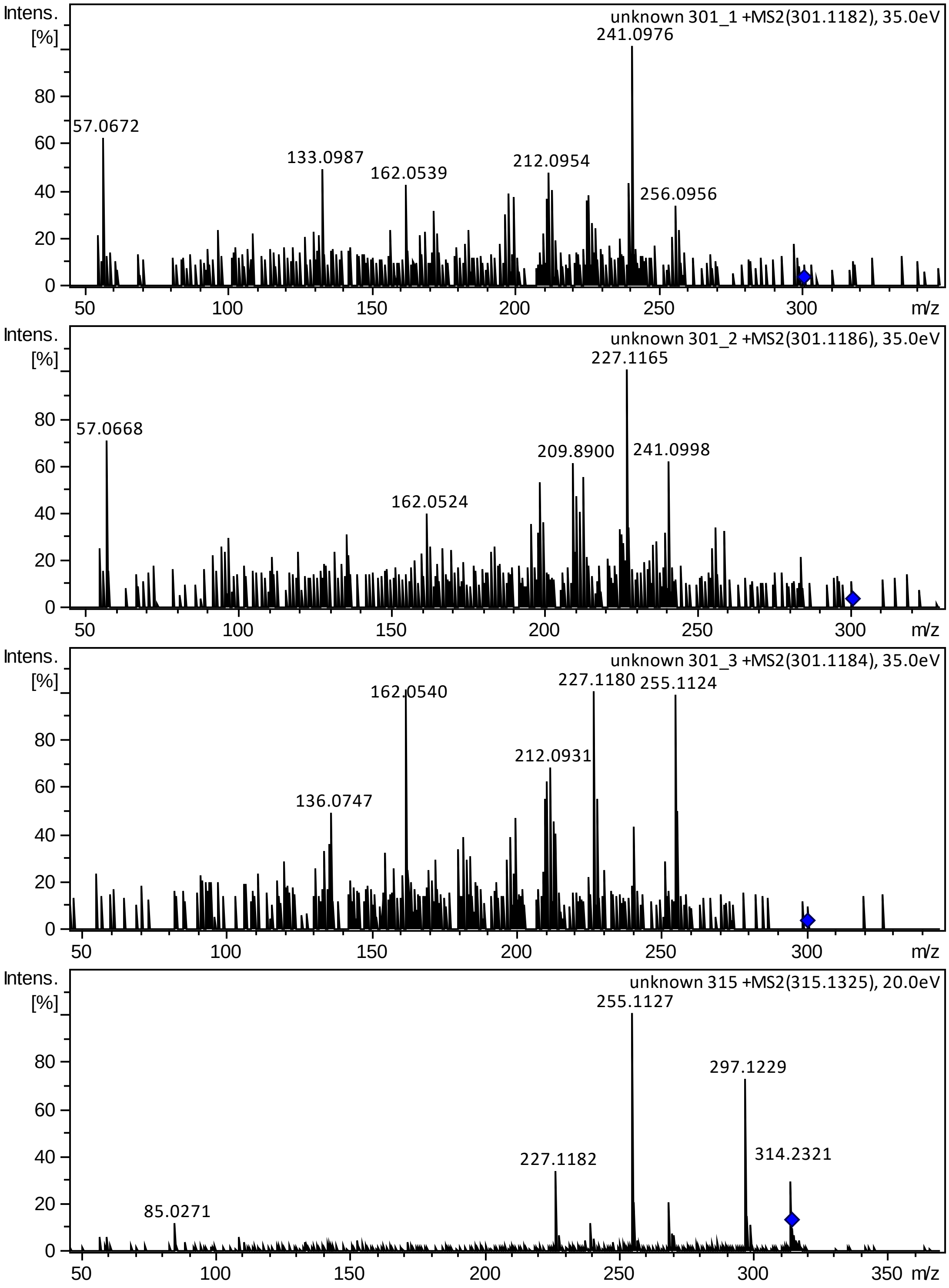

Figure S6. High resolution MS/MS spectra of unknown breakdown products in positive ESI mode. The precursor ion is marked with a blue pane.

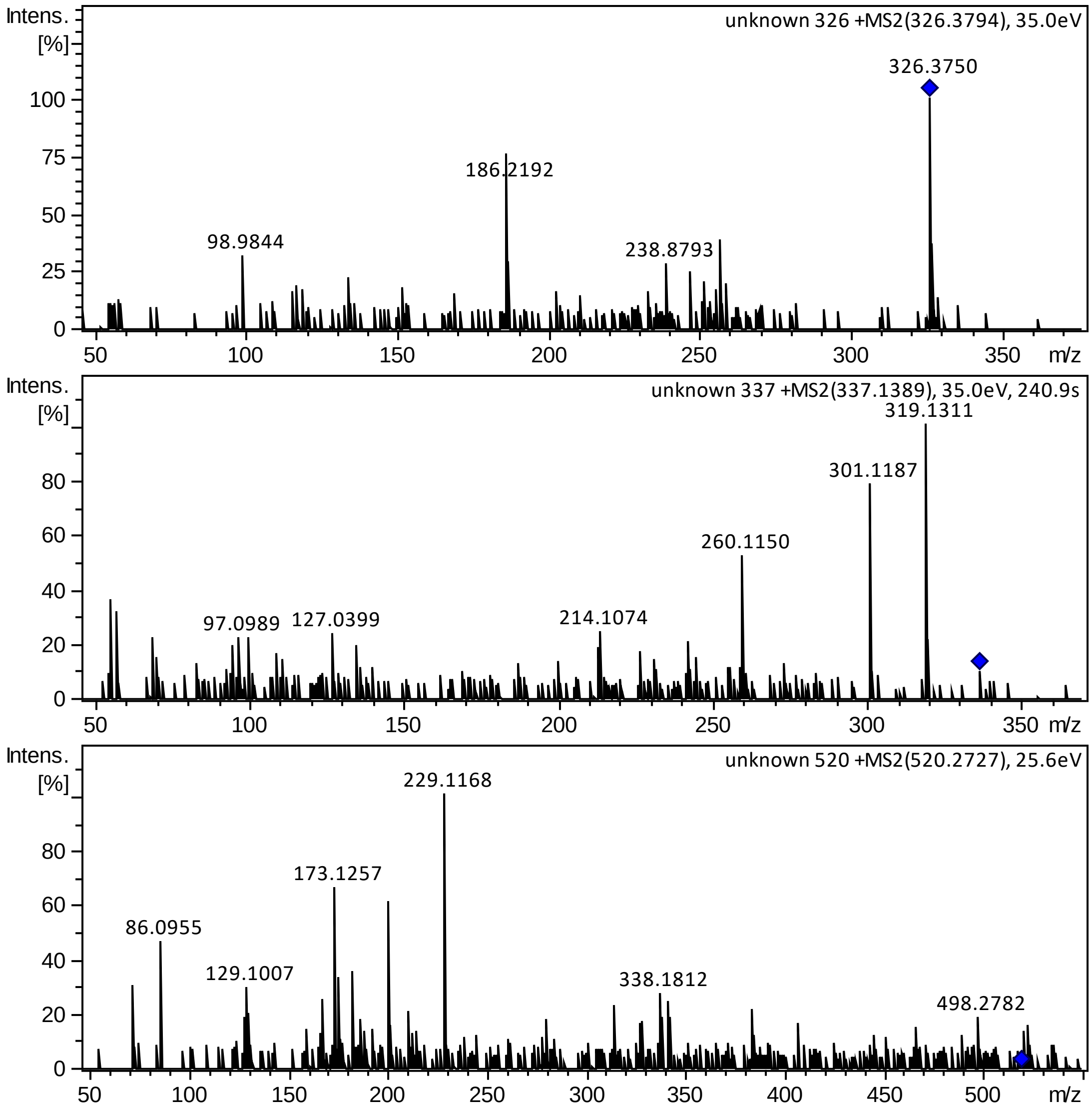

Figure S6 (continued). High resolution MS/MS spectra of unknown breakdown products in positive ESI mode. The precursor ion is marked with a blue pane.

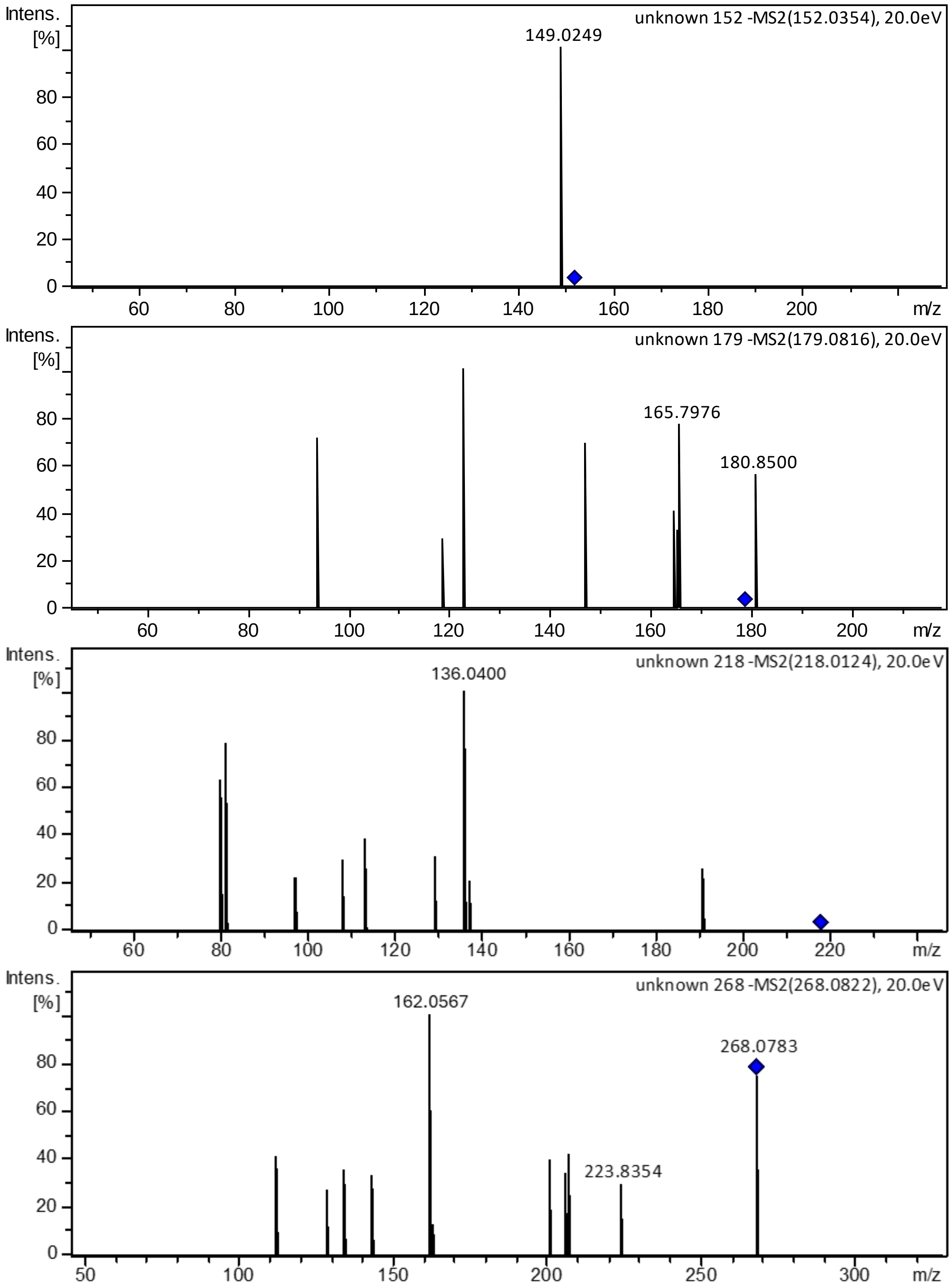

Figure S7. High resolution MS/MS spectra of unknown breakdown products in negative ESI mode. The precursor ion is marked with a blue pane.

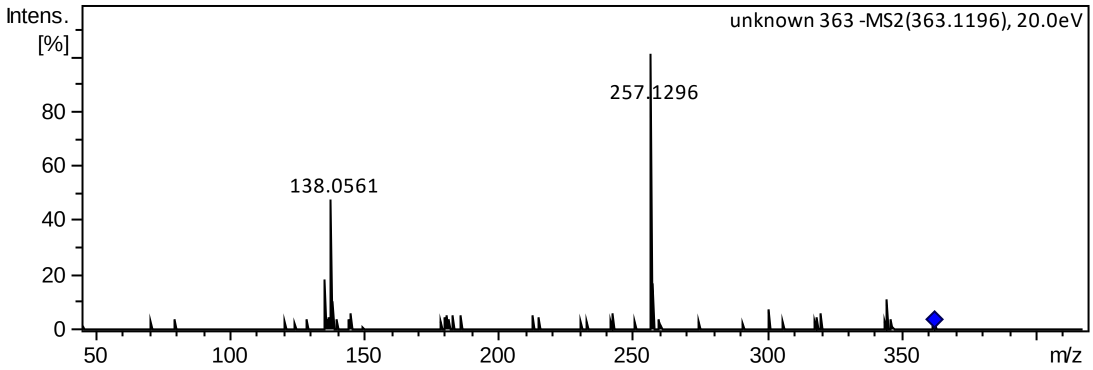

Figure S7 (continued). High resolution MS/MS spectra of unknown breakdown products in negative ESI mode. The precursor ion is marked with a blue pane.

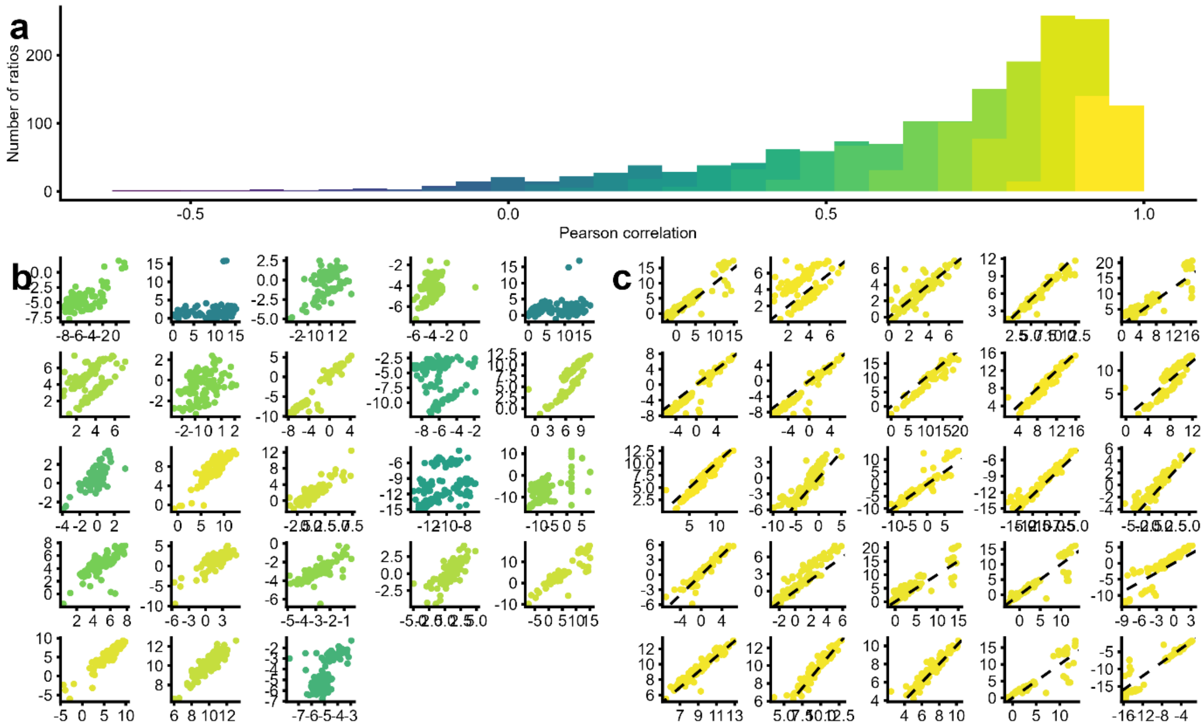

Figure S8. Filtering of ratios using technical replicates. (**a**) Distribution of person correlations between all pairs of metabolite ratios. (**b**) Scatterplot of replicate 1 values (x) and replicate 2 values (y) from 23 random ratios. (**c**) Scatterplot of replicate 1 values (x) and replicate 2 values (y) from 25 random ratios with high correlation.

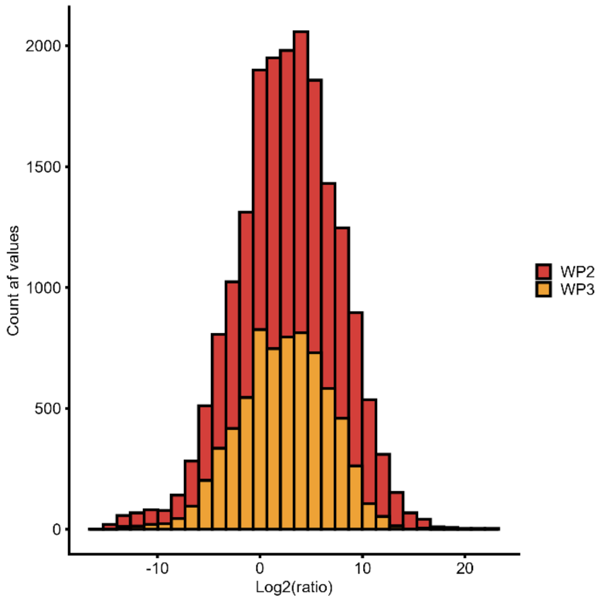

Figure S9. Distribution of log2(ratio) values in the targeted training data.

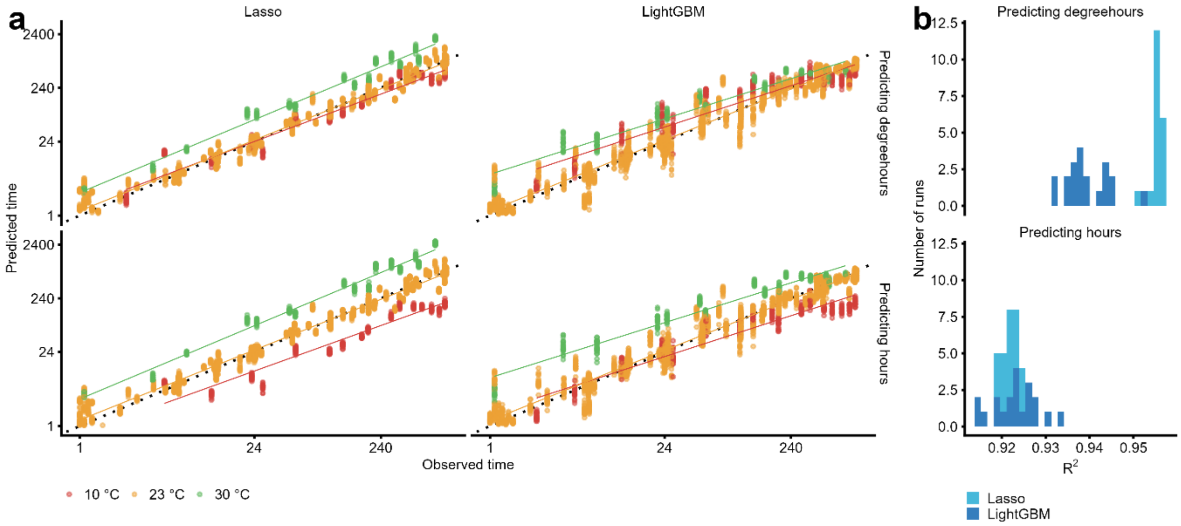

Figure S10. Effect of temperature and using either hours or degreehours as response. (**a**) Observed and predicted hours using either hours (bottom) or degree hours (top) as response for two machine learning methods when only training on 23°C samples. (**b**) R^2 values from 20 independent runs using Lasso (top) and LightGBM (bottom) on either degreehours (light blue) or hours (dark blue).

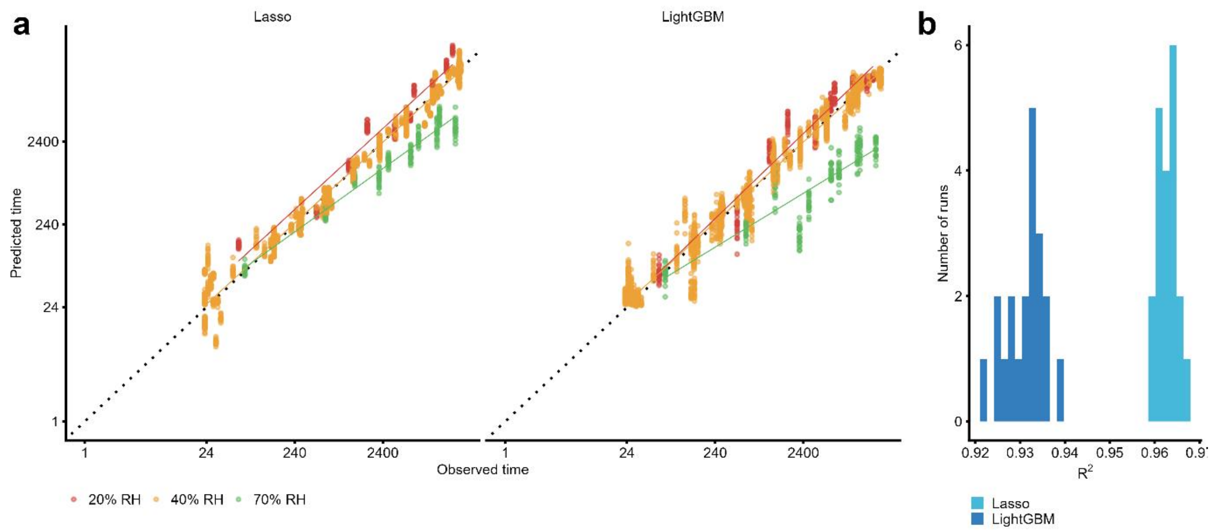

Figure S11. Effect of humidity. (**a**) Observed and predicted time since deposition as response for two machine learning methods (using degreehours) for different relative humidities, when only trained on 40% RH. (**b**) R^2 values from 20 independent runs using Lasso (light blue) and LightGBM (dark blue).

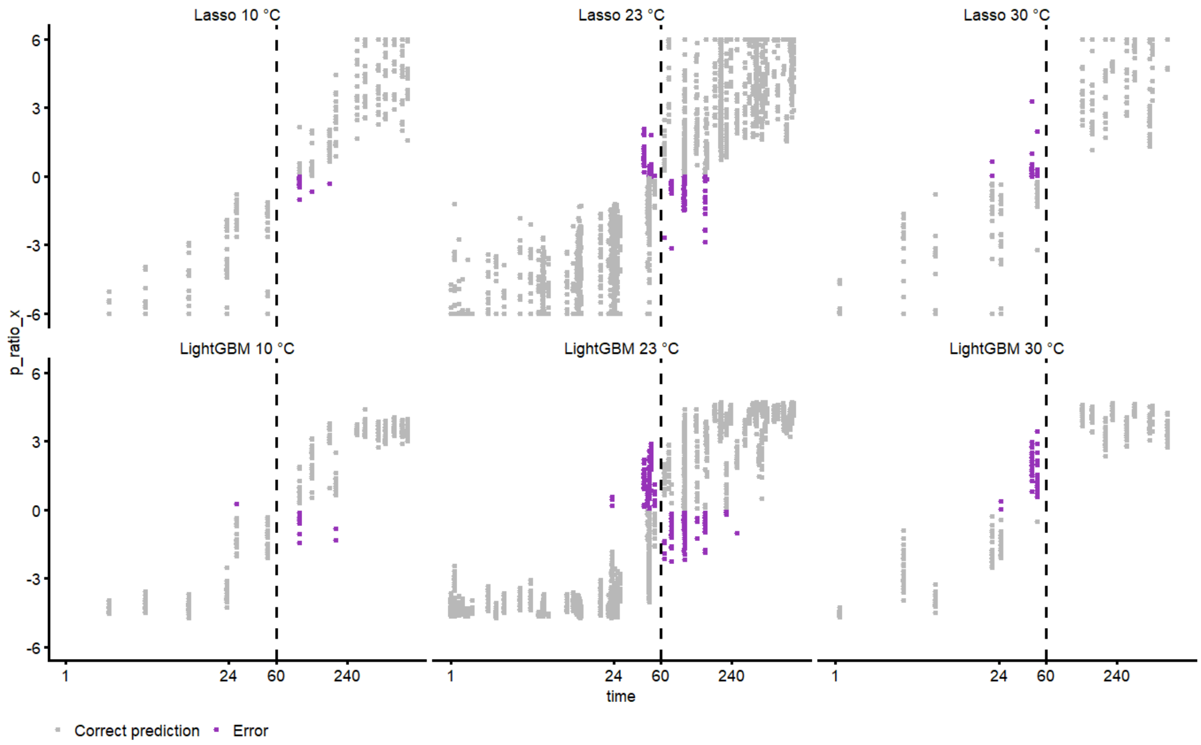

Figure S12. Cross-validated Log-odds for different timepoints for 20 independent runs. Misclassifications were close to the 60-hour threshold and showed low confidence (absolute log-odds scores close to 0) whereas extremely fresh or old samples were typically far from the threshold and showed high confidence (high absolute log-odds scores).

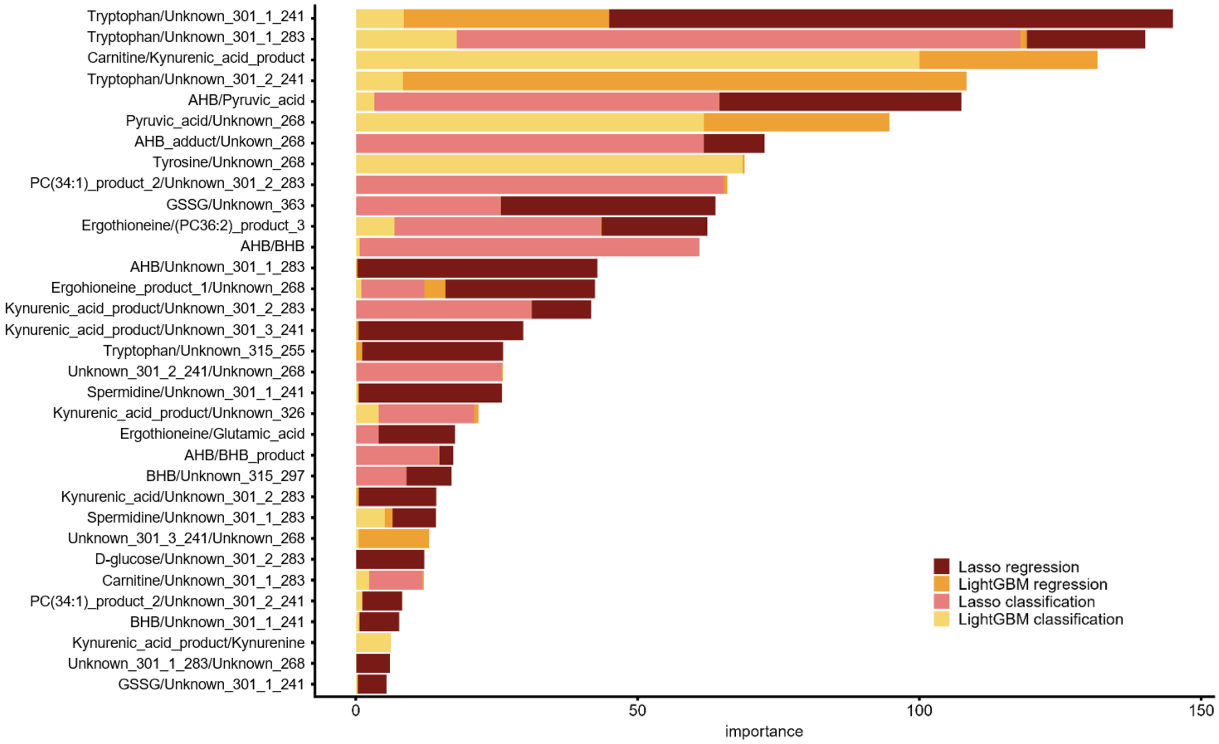

Figure S13. Ratio importance measures from different models and objectives (Lasso and LightGBM and regression and classification. The most important feature from each model and objective is scaled to 100 and only ratios with at least one importance > 5 is shown. Generally the importance quickly degrades and only a few ratios are needed for the models.

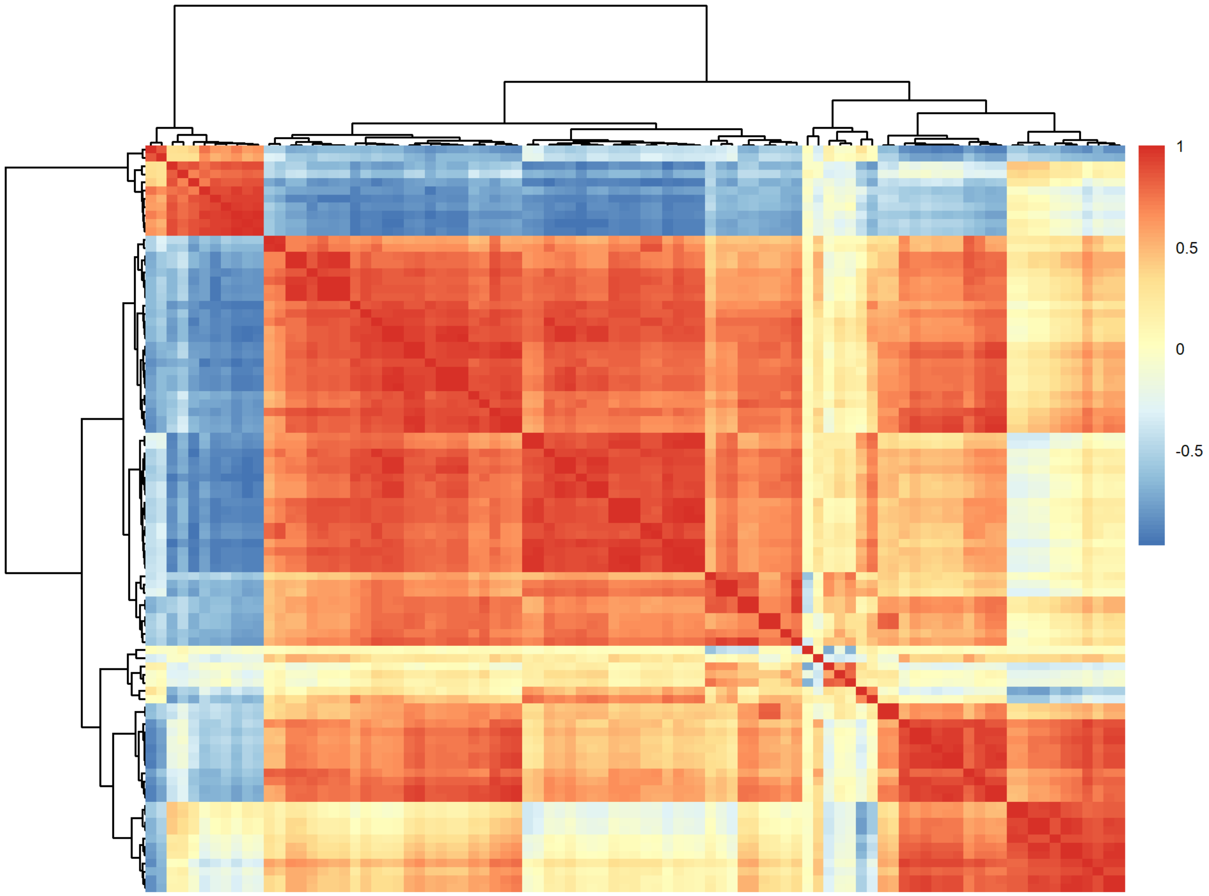

Figure S14. Correlation heatmap using spearman rank correlation of the 91 ratios showing large clusters of highly correlated ratios.

Table S1. Donor characteristics, sampling practice, and bloodstain aging conditions for the different datasets. The targeted analysis set included both new samples (described in the table) and the samples from the untargeted analysis for machine learning modelling.

	Untargeted analysis set	Targeted analysis set	Validation set #1	Validation set #2
**Number of donors (Females/males)**	1 / 4	36 / 17	3 / 1	2 / 2
**Donor age, years (mean ± SD)**	34.6 ± 8.8	39.9 ± 11.6	Not specified	Not specified
**Blood sampling per donor**	30	1-7	1	1
**Samples per donor**	30<br>(3 per time group)	1-7	5<br>(1 per time group)	5-8
**Duration of bloodstain aging (time groups)**	1h<br>6h<br>12h<br>24h<br>2 days<br>4 days<br>8 days<br>16 days<br>32 days<br>64 days	Random from 1h to 49 days	1h<br>24h<br>2 days<br>3 days<br>8 days	Random from 2h to 17 days
**Total number of samples**	150	79<br>(+ 150 from untargeted analysis)<br>Total 229	20	26
**Blood sampling practice**	Fingertip needle prick	Fingertip needle prick	Venipuncture	Venipuncture
**Sample volume, µL**	50	Approx. 10 to 50 µL	50	50
**Aging conditions**	23°C and 40% humidity	1. 30°C and 40% humidity (n=16)<br>2. 10°C and 40% humidity (n=17)<br>3. 23°C and 70% humidity (n=10)<br>4. 23°C and 20% humidity (n=10)<br>5. indoor mock-up samples (n=26)	23°C and 40% humidity	Indoor mock-up samples, temperature at sampling was measured

Table S2. List of components for the targeted LC-MS/MS method including MRM transitions and retention times. Compounds included in our standard mix (17 components) are marked with an asterisk*. Internal standards are marked IS.

**Component**	MRM transitions		**RT**	**Ionization**	DP (V)	EP (V)	CE (eV)	CXP (V)
	**Q1 (*m/z*)**	**Q3 (*m/z*)**	**(min)**	**mode**				
**Tryptophan**	205.1	146.0	5.15	Pos	10	10	24	17
**Kynurenine***	209.1	120.1	4.10	Pos	20	10	33	13
**Kynurenine product 1**	146.0	77.0/91.0	4.95	Pos	130	10	36	10
**Kynurenine product 2**	215.1	141.0/169.1	4.69	Pos	10	11	27	10
**Kynurenic acid***	190.1	116.1	5.52	Pos	40	10	43	14
**Kynurenic acid product**	120.0	65.0/92.0	6.00	Pos	20	7	21	12
**D-Glucose*/fructose**	178.7	135.0	0.54	Neg	-30	-10	-16	-9
**Ergothioneine***	230.0	127.1	0.84	Pos	28	10	25	14
**Ergothioneine product 1 (hercynine)**	198.1	68.0/95.1	0.69	Pos	30	10	27	10
**Ergothioneine product 2**	229.1	185.1/229.1	1.46	Pos	16	10	19	12
**Cystine***	241.0	152.1	0.67	Pos	18	10	18	20
**Palmitoyl carnitine**	400.0	239.1	10.50	Pos	45	5	27	10
**Carnitine***	162.1	103.0	0.71	Pos	30	14	22	11
**3-hydroxybutyric acid (BHB)***	103.0	58.9	2.00	Neg	-25	-12	-13	-9
**BHB product**	117.0	73.0/99.0	1.70	Neg	-15	-10	-16	-7
**Glutamic acid***	148.0	84.0	0.69	Pos	10	10	22	10
**Citric acid***	191.0	111.0	1.20	Neg	-20	-10	-17	-12
**Pyruvic acid***	87.0	43.1	0.87	Neg	-17	-14	-11	-5
**Tyrosine***	182.1	136.1	1.80	Pos	20	10	18	10
**Spermidine***	146.1	72.0	0.54	Pos	20	13	19	10
**Spermidine product**	184.1	85.0	2.25	Pos	40	11	26	11
**Ox. Glutathione (GSSG)***	613.1	355.0	2.20	Pos	90	10	32	15
**Red. Glutathione (GSH)***	308.1	179.2	0.99	Pos	40	10	16	9
**NAD^{+}***	664.1	428.0/524.0	1.38	Pos	60	9	35	25
**Niacinamide**	123.0	80.0	1.20	Pos	50	9	30	20
**2-hydroxybutyric acid (AHB)***	103.0	57.0	2.49	Neg	-31	-6	-14	-7
**AHB adduct**	171.0	103.0	2.49	Neg	-45	-10	-16	-10
**Sebacic acid**	201.0	139.0	7.69	Neg	-34	-10	-23	-11

**Sphingosine-1-phosphate**	378.2	78.9	10.89	Neg	-100	-10	-80	-10
**Sphinganine-phosphate**	380.2	78.9	11.45	Neg	-70	-13	-95	-8
**PC34:1(16:0/18:1)**	760.5	184.0	16.71	Pos	20	10	40	9
**PC34:1 product 1**	666.4	184.0/297.3	11.87	Pos	40	10	45	15
**PC34:1 product 2**	650.4	184.0	12.67	Pos	60	5	41	10
**PC34:1 product 3**	776.6	184.0	13.85	Pos	60	7	42	20
**LysoPC16:0**	496.3	184.0	11.90	Pos	110	8	35	20
**PC36:2(18:0/18:2)**	786.4	184.0	17.79	Pos	35	10	40	11
**PC36:2 product 1**	428.2	349.0	5.70	Pos	60	12	22	20
**PC36:2 product 2**	678.5	184.0	12.78	Pos	60	8	36	10
**PC36:2 product 3**	818.6	184.0	13.70	Pos	40	9	44	10
**Unknown 301_1**	301.1	241.1/283.0	7.10	Pos	50	7	20	20
**Unknown 301_2**	301.1	241.1/283.0	7.29	Pos	50	7	20	20
**Unknown 301_3**	301.1	241.1/283.0	7.53	Pos	40/50	10	26/20	14/20
**Unknown 315**	315.1	255.1/297.1	8.10	Pos	10/50	10	20	13
**Unknown 326**	326.4	303.3	9.50	Pos	60	5	15	20
**Unknown 337**	337.0	219.2	4.44	Pos	13	7	16	15
**Unknown 520**	520.3	308.1/498.3	5.57	Pos	15	9	14	24
**Unknown 152**	152.0	149.0	4.38	Neg	-20	-10	-15	-10
**Unknown 179**	179.1	165.8	4.87	Neg	-20	-10	-5	-10
**Unknown 218**	218.0	136.0	4.86	Neg	-20	-10	-5	-10
**Unknown 268**	268.1	162.1	6.71	Neg	-20	-10	-25	-10
**Unknown 363**	363.1	257.0	6.03	Neg	-20	-5	-22	-16
**L-Glutamic acid-$^{13}C_5$ (IS)**	153.0	88.3	0.68	Pos	30	4.5	21	15
**L-Tryptophan D_8 (IS)**	213.1	195.2	5.10	Pos	20	4	15	15
**DL-A-phosphatidylcholine. dipalmitoyl (U-$^{13}C_{40}$) (IS)**	774.6	189.0	15.80	Pos	40	13.5	38.5	25
